# Clinical Exome Sequencing in Unexplained Hyperferritinemia Reveals Digenic and Oligogenic Inheritance Beyond Iron Homeostasis

**DOI:** 10.1111/liv.70646

**Published:** 2026-04-12

**Authors:** Paul Morel, Maël Silva Rodriguez, Cyriaque Benmouffek, Betul Goksen, Céline Chéry, Vincent Haghnejad, Mouni Bensenane, François Feillet, Jean‐Louis Guéant, Farès Namour, Jean‐Pierre Bronowicki, Abderrahim Oussalah

**Affiliations:** ^1^ Department of Genomic Medicine, Division of Biochemistry, Molecular Biology, and Nutrition University Hospital of Nancy Nancy France; ^2^ Reference Center for Inborn Errors of Metabolism (ORPHA67872) University Hospital of Nancy Nancy France; ^3^ Faculty of Medicine of Nancy University of Lorraine Nancy France; ^4^ INSERM UMR_S 1256, Nutrition, Genetics, and Environmental Risk Exposure (NGERE) Nancy France; ^5^ Department of Gastroenterology and Liver Diseases University Hospital of Nancy Nancy France; ^6^ Department of Paediatrics University Hospital of Nancy Nancy France; ^7^ Joan Klein Jacobs Center for Precision Nutrition and Health Cornell University Ithaca New York USA

**Keywords:** clinical exome sequencing, digenic inheritance, genotype–phenotype correlation, hemochromatosis, hyperferritinemia, iron overload

## Abstract

**Background and Aims:**

Hyperferritinemia encompasses heterogeneous genetic etiologies beyond *HFE*‐related hemochromatosis. Current guidelines recommend testing for rare hemochromatosis genes, yet no consensus exists on comprehensive genomic approaches. We aimed to characterise the genetic landscape of unexplained hyperferritinemia using clinical exome sequencing (CES) and evaluate genotype–phenotype correlations across functional pathways.

**Methods:**

In this retrospective study (2019–2024), consecutive patients with unexplained hyperferritinemia after exclusion of secondary causes underwent CES. Patients with known *HFE* p.Cys282Tyr homozygosity were not referred for CES. Variant filtering was performed using the Genomics England panel for iron metabolism and the French network for rare liver diseases panel, combined with phenotype‐driven analysis based on Human Phenotype Ontology annotations for iron‐related phenotypes. Genes were categorised into four pathways: systemic iron sensing, iron transport and storage, hepatic metabolism and erythropoiesis.

**Results:**

Among 108 patients, CES identified at least one variant in 72 (66.7%), including 44 (40.7%) with likely pathogenic or pathogenic (LP/P) variants. *HFE* was the most frequently affected gene, followed by *SERPINA1*, *ATP7B*, and *CP*. Among patients without monogenic *HFE* alterations (*n* = 57), 21 (36.8%) carried variants, mainly affecting *CP*, *ATP7B*, *SERPINA1* or *GBA*. Digenic or oligogenic inheritance was observed in 30.6% (22/72) of patients overall and in 20.5% (9/44) of those carrying LP/P variants, most frequently involving *HFE*‐*SERPINA1* and *HFE*‐*ATP7B* combinations. Cross‐pathway combinations occurred in 17 of 22 digenic patients (77.3%). The systemic iron sensing group exhibited significantly higher serum iron (*p* = 0.009) and transferrin saturation (*p* = 0.008).

**Conclusions:**

CES reveals genetic heterogeneity beyond the traditional Mendelian framework, with frequent non‐*HFE* gene involvement and digenic inheritance and should be considered after exclusion of *HFE* p.Cys282Tyr homozygosity.

## Lay Summary

When blood ferritin levels are elevated without a clear explanation, doctors typically test for a single common gene linked to iron overload, but many patients remain without a diagnosis. Using broad genetic testing that examines approximately 6700 genes, this study of 108 patients found that more than 40% carried disease‐causing variants, often in genes related to liver metabolism or blood cell production rather than classical iron overload, and that nearly one‐third had variants in two or more genes acting together. These results support the use of comprehensive genetic testing after ruling out the most common form of hereditary hemochromatosis, enabling earlier identification of treatable conditions and more personalised patient care.

**Abbreviations**
ACMG: American College of Medical Genetics and Genomics
ALT: alanine aminotransferase
AST: aspartate aminotransferase
CES: clinical exome sequencing
ERYTHRO‐HEME: erythropoiesis and heme biosynthesis
GGT: gamma‐glutamyltransferase
HEPATO‐METAB: hepatic metabolism and lysosomal storage
HGVS: Human Genome Variation Society
HIC: hepatic iron concentration
HMZ: homozygous
HTZ: heterozygous
IQR: interquartile range
IRON‐SENS: systemic iron sensing and hepcidin regulation
IRON‐TRANS: iron transport and storage
LP: likely pathogenic
NGS: next‐generation sequencing
P: pathogenic
VUS: variant of uncertain significance

## 1 Introduction

Hyperferritinemia is a frequent laboratory finding in primary care, with a prevalence ranging from 5.3% to 25.4% in apparently healthy individuals depending on ethnicity, as reported in the Hemochromatosis and Iron Overload Screening (HEIRS) Study [1]. While most cases are attributable to acquired causes such as inflammation, hepatic cytolysis, excessive alcohol consumption, or metabolic dysfunction, a subset reflects underlying genetic disorders [2, 3]. Although *HFE*‐related hemochromatosis remains the predominant form in European populations, rare hemochromatosis genes account for a genetically heterogeneous subset of cases [4, 5, 6]. Current guidelines recommend testing for rare hemochromatosis genes to provide diagnostic certainty and to enable family screening [4]. In clinical practice, hyperferritinemia is also observed in patients with hereditary hepatic and lysosomal storage disorders, yet these conditions are not systematically considered in current diagnostic algorithms.

Clinical exome sequencing (CES) enables comprehensive interrogation of protein‐coding regions across thousands of genes in a single assay [7, 8, 9]. Exome and whole‐genome sequencing have been evaluated for use in genetic disorders, with more evidence for CES, exhibiting a molecular diagnostic yield ranging from 9% to 51% [8]. Previous studies evaluating next‐generation sequencing in iron metabolism disorders have primarily employed targeted gene panels comprising 5 to 39 genes and focused on patients with confirmed iron overload or suspected hemochromatosis [10, 11, 12, 13, 14, 15, 16, 17]. However, the diagnostic performance of CES in the broader population of patients with unexplained hyperferritinemia, encompassing genetic etiologies beyond classical hemochromatosis genes, has not been systematically evaluated. In hyperferritinemia, CES offers the potential to simultaneously investigate genes across multiple pathophysiological mechanisms.

We conducted a retrospective study of consecutive patients who underwent CES for the investigation of unexplained hyperferritinemia at a tertiary referral center in routine clinical practice. Our aims were to determine the diagnostic yield of CES, characterise the genetic landscape across functional pathways, evaluate the prevalence of digenic and oligogenic inheritance patterns, and assess genotype–phenotype correlations with biochemical iron parameters and hepatic iron concentration measured by magnetic resonance imaging.

## 2 Patients and Methods

### 2.1 Study Design and Patient Selection

We conducted a retrospective observational study of consecutive patients referred to the Reference Center for Inborn Errors of Metabolism at the University Hospital of Nancy (France) for CES between January 2019 and December 2024. Prior to genetic testing, patients underwent clinical and biochemical evaluation by expert hepatologists to assess for secondary causes of hyperferritinemia, particularly systemic inflammation, hepatic cytolysis, metabolic dysfunction‐associated steatotic liver disease, excessive alcohol consumption, and iron‐loading anemias. CES was performed when hyperferritinemia remained unexplained or disproportionate to identified secondary factors. Patients were included if they had a clinical indication of unexplained hyperferritinemia and met at least one of the following criteria: (1) elevated serum ferritin concentration (greater than 300 μg/L in males or greater than 200 μg/L in females); (2) elevated transferrin saturation (greater than 50% in males or greater than 45% in females); (3) elevated hepatic iron concentration by magnetic resonance imaging (greater than 36 μmol/g); or (4) clinical suspicion of iron metabolism disorder based on physician clinical judgement, in patients with borderline biochemical values not meeting the above thresholds and a prior history of iron overload documented on routine laboratory assessment of iron metabolism parameters. Sex‐specific thresholds for serum ferritin and transferrin saturation were defined according to European Association for the Study of the Liver guidelines [4]. Clinical exome sequencing was not performed in patients with: (1) known homozygosity for p.Cys282Tyr in *HFE*, given the well‐established genotype–phenotype correlation for this condition; or (2) hyperferritinemia fully explained by secondary causes identified during clinical and biochemical evaluation. Details on the French framework for genetic diagnosis, ethics approval, data collection procedures, and data availability are provided in the Supporting Information [8, 9].

### 2.2 Next‐Generation Sequencing

CES was performed in accordance with ISO 15189 in compliance with the French Accreditation Committee requirements on DNA extracted from peripheral blood leukocytes using the TruSight One Expanded panel (approximately 6700 genes; Table S1) on an Illumina NextSeq 550 platform at the Department of Genomic Medicine, University Hospital of Nancy. Variant filtering used the Genomics England PanelApp panel for iron metabolism disorders (27 genes) and the French national network for rare liver diseases panel FILFOIE (183 genes). Phenotype‐driven prioritisation was based on four Human Phenotype Ontology annotations: increased circulating ferritin concentration (HP:0003281, 58 genes), elevated transferrin saturation (HP:0012463, 11 genes), elevated hepatic iron concentration (HP:0012465, 12 genes) and abnormality of iron homeostasis (HP:0011031, 30 genes). After exclusion of shared genes, the total number of genes of interest was 251 (Table S1). Variants were classified according to American College of Medical Genetics and Genomics criteria under the sole responsibility of clinical molecular geneticists. Detailed information on the bioinformatics pipelines is provided in the Supporting Information.

### 2.3 Functional Classification of Genes

All variants reported in the molecular diagnosis reports as part of routine clinical care were extracted for this study. Genes harbouring at least one variant classified as VUS, LP or P were then classified a posteriori into four functional groups according to their roles in iron homeostasis and the pathophysiological mechanisms through which they contribute to hyperferritinemia: IRON‐SENS (systemic iron sensing and hepcidin regulation: *HFE*, *HJV*, *HAMP*, *TFR2*), IRON‐TRANS (iron transport and storage: *TF*, *FTL*, *CP*, *ATP7B*), HEPATO‐METAB (hepatic metabolism and lysosomal storage: *SERPINA1*, *GBA*, *CFTR*, *HNF1B*, *BCS1L*) and ERYTHRO‐HEME (erythropoiesis and heme biosynthesis: *HBB*, *ANK1*, *SLC4A1*, *UROD*). The detailed rationale for this classification is provided in the Supporting Information.

### 2.4 Study Aims

The primary aim of this study was to characterise the genetic landscape of patients with unexplained hyperferritinemia who underwent clinical exome sequencing. Secondary aims included evaluating the distribution of genetic variants across the four functional pathways defined above, characterising patterns of digenic and oligogenic architecture, and assessing genotype–phenotype correlations with iron metabolism parameters.

### 2.5 Study Outcomes and Endpoints

We assessed the following outcomes: the number of genetic variants identified per patient; the distribution of variants according to gene, zygosity (heterozygous, homozygous or double heterozygous [cis or trans]) and ACMG classification (VUS, LP, or P); patterns of gene co‐occurrence among patients harbouring variants in multiple genes, categorised as monogenic, digenic or oligogenic architecture; and the distribution of patients according to functional pathway classification.

The primary endpoints were the proportion of patients carrying at least one pathogenic variant (LP or P) and the proportion of patients carrying at least one clinically retained variant (VUS, LP or P). Secondary endpoints were: (i) the proportion of patients exhibiting digenic or oligogenic architecture (defined as the co‐occurrence of variants in three or more distinct genes); and (ii) differences in iron metabolism parameters (serum ferritin, serum iron, transferrin, transferrin saturation and hepatic iron concentration measured by magnetic resonance imaging) across the four functional pathway groups.

### 2.6 Statistical Analysis

Continuous variables are expressed as medians with interquartile ranges. This study was exploratory, and no a priori sample size calculation was performed. *p*‐values are reported for descriptive purposes without adjustment for multiple comparisons. Comparisons across functional pathway groups were performed using the Kruskal–Wallis test, with effect sizes estimated using eta‐squared. Pathway comparison analyses were conducted including all retained variants (VUS, LP or P) and restricted to LP or P variants only. Detailed statistical methods are provided in the Supporting Information. All analyses were performed using Python version 3.12.

## 3 Results

### 3.1 Study Population

Between January 2019 and December 2024, 108 patients underwent CES for the investigation of unexplained hyperferritinemia. The study population comprised predominantly men (87 [80.6%]) with a median age of 56.5 years (interquartile range [IQR], 41.8–65.0 years) (Table 1). Median serum ferritin was 544 μg/L (IQR, 339–807) and transferrin saturation was 42.0% (IQR, 30.0–53.0). Median hepatic iron concentration measured by magnetic resonance imaging was 52.0 μmol/g (IQR, 38.0–97.0), indicating that at least 75% of patients exceeded the upper limit of normal (36 μmol/g). Median C‐reactive protein was 4.0 mg/L (IQR, 4.0–4.0), indicating absence of systemic inflammation.

**TABLE 1:** Baseline characteristics of patients referred for clinical exome sequencing for unexplained hyperferritinemia.

Characteristic	Total (*N* = 108)
Demographics	
Age, median (IQR), y	56.5 (41.8–65.0)
Turnaround time, median (IQR), mo	6.00 (4.00–10.00)
Sex, No. (%)	
Male	87 (80.6)
Female	21 (19.4)
Comorbidities and alcohol use, No. (%)	
Comorbidities^a^	11 (10.2)
Alcohol consumption	
Abstinent	48 (44.4)
Occasional	33 (30.6)
Low	16 (14.8)
Moderate	6 (5.6)
High	5 (4.6)
Anthropometric measurement, median (IQR)	
Body mass index, kg/m^2^	26.2 (24.0–29.3) [*n* = 98]
Iron metabolism, median (IQR)	
Serum ferritin, μg/L [M: 22–322; F: 10–291]^b^	544 (339–807) [*n* = 96]
Serum iron, mg/L [M: 0.65–1.75; F: 0.5–1.7]^b^	1.18 (0.89–1.50) [*n* = 95]
Transferrin, g/L [M: 2.15–3.65; F: 2.5–3.8]^b^	2.00 (1.80–2.30) [*n* = 92]
Transferrin saturation, % [20–40]^b^	42.0 (30.0–53.0) [*n* = 94]
Liver function tests, median (IQR)	
ASAT, U/L [13–40]^b^	27.5 (22.0–41.8) [*n* = 94]
ALAT, U/L [7–40]^b^	32.5 (20.0–54.5) [*n* = 94]
GGT, U/L [M: ≤ 73; F: ≤ 38]^b^	36.5 (22.0–85.8) [*n* = 90]
Alkaline phosphatase, U/L [46–116]^b^	72.0 (58.0–86.0) [*n* = 93]
Albumin, g/L [35–52]^b^	42.7 (40.1–44.7) [*n* = 80]
Prothrombin time, % [70–100]^b^	98.0 (92.8–100.0) [*n* = 80]
INR [2–3]^b^	1.10 (1.10–1.20) [*n* = 11]
Total protein, g/L [57–82]^b^	72.0 (69.5–75.0) [*n* = 55]
Metabolic parameters, median (IQR)	
Fasting glucose, g/L [0.74–1.06]^b^	0.90 (0.81–1.04) [*n* = 86]
Triglycerides, g/L [< 1.5]^b^	1.30 (0.99–2.21) [*n* = 77]
Total cholesterol, g/L [Desirable, < 2.0]^b^	1.99 (1.65–2.17) [*n* = 77]
Inflammatory marker, median (IQR)	
C‐reactive protein, mg/L [< 3.3]^b^	4.00 (4.00–4.00) [*n* = 81]
Hematologic parameters, median (IQR)	
Haemoglobin, g/dL [M: 13.5–17.5; F: 12.0–16.0]^b^	14.7 (13.8–15.5) [*n* = 94]
MCV, fL [80–100]^b^	91.6 (88.4–95.8) [*n* = 94]
Platelet count, ×10^9^/L [150–450]^b^	233 (185–273) [*n* = 92]
WBC count, ×10^9^/L [4–10]^b^	6.43 (5.30–7.75) [*n* = 94]
Renal function, median (IQR)	
Serum creatinine, mg/L [M: 7.0–13.0; F: 5.5–10.2]^b^	7.90 (7.10–9.30) [*n* = 95]
Blood urea, g/L [0.19–0.49]^b^	0.29 (0.26–0.36) [*n* = 91]
eGFR (CKD‐EPI), mL/min/1.73 m^2^ [> 90]^b^	97.8 (87.0–106.8) [*n* = 94]
Copper metabolism, median (IQR)	
Serum copper, mg/L [0.70–1.50]^b^	0.92 (0.77–1.01) [*n* = 43]
Hepatic imaging, median (IQR)	
Hepatic iron concentration, μmol/g [5–27]^b^	52.0 (38.0–97.0) [*n* = 25]
Liver stiffness (FibroScan), kPa [< 5]^b^	5.40 (4.30–6.80) [*n* = 69]

*Note:* Continuous variables are presented as median (interquartile range); [*n* = X] indicates the number of patients with available data when less than the total. Categorical variables are presented as No. (%).

Abbreviations: ALAT, alanine aminotransferase; ASAT, aspartate aminotransferase; CKD‐EPI, chronic kidney disease epidemiology collaboration; eGFR, estimated glomerular filtration rate; F, female; GGT, gamma‐glutamyltransferase; INR, international normalised ratio; IQR, interquartile range; M, male; MCV, mean corpuscular volume; WBC, white blood cell.

^a^ Comorbidities included metabolic syndrome (1 [0.9%]), active viral hepatopathy (1 [0.9%]), chronic kidney disease (3 [2.8%]), chronic inflammatory disease (1 [0.9%]), hemolytic disorder (1 [0.9%]), chronic anaemia (2 [1.9%]), iron supplementation (1 [0.9%]), and erythropoietin therapy (1 [0.9%]). No patient had history of chronic transfusion.

^b^ Reference values are adapted to an adult population.

### 3.2 Diagnostic Yield and Genetic Findings

**FIGURE 1:**
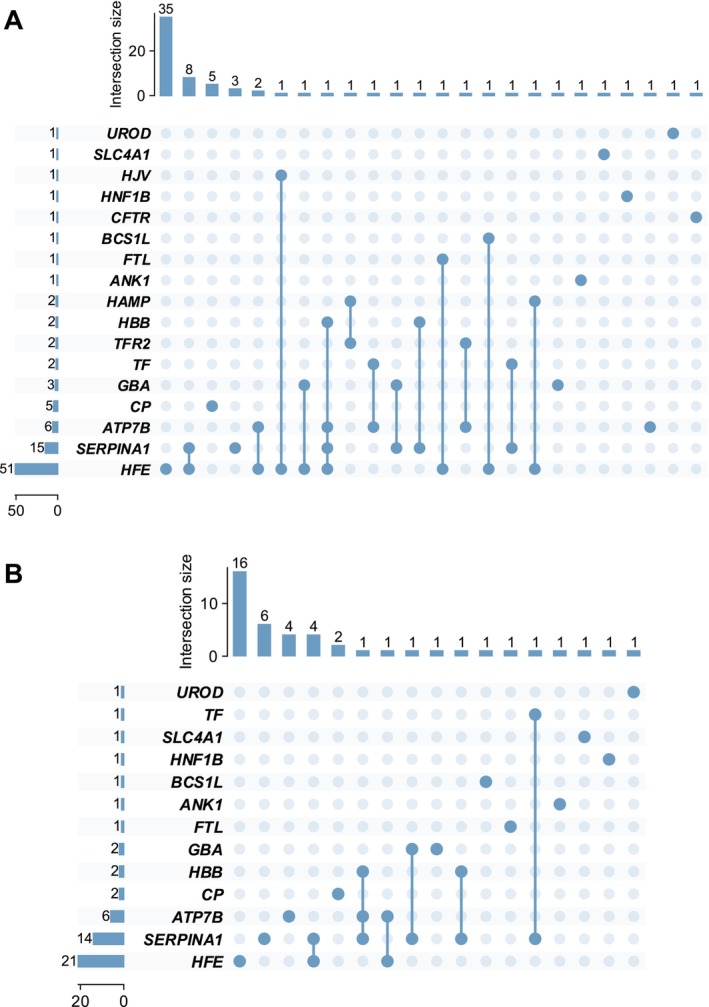
Gene co‐occurrence patterns in patients with hyperferritinemia. (A) UpSet plot depicting gene co‐occurrence patterns among 72 patients harbouring at least one variant classified as VUS, LP or P according to American College of Medical Genetics and Genomics criteria (all retained variants). LP, likely pathogenic; P, pathogenic; VUS, variant of uncertain significance. Horizontal bars on the left indicate the total number of patients with variants in each gene. Vertical bars represent the intersection size for each unique gene combination. Single dots indicate involvement of a single gene (including patients with two variants in the same gene), whereas connected dots denote digenic or oligogenic patterns involving multiple genes. The most frequently involved genes were *HFE* (*n* = 51), *SERPINA1* (*n* = 15), *ATP7B* (*n* = 6), and *CP* (*n* = 5). The largest subset comprised 35 patients with *HFE* variants only, followed by eight patients with *SERPINA1* only. (B) UpSet plot restricted to patients with LP or P variants only (*n* = 44). Among these patients, *HFE* remained the most commonly affected gene (*n* = 21), followed by *SERPINA1* (*n* = 14) and *ATP7B* (*n* = 6). The largest subset comprised 16 patients with *HFE* variants only, followed by six patients with *SERPINA1* only and four patients with *HFE* and *SERPINA1* co‐occurrence.

CES identified at least one genetic variant (VUS, LP or P) in 72 of 108 patients (66.7%). At least one LP or P variant was identified in 44 patients (40.7%), representing the primary endpoint of the study. Among the 72 patients with variants, 44 (61.1%) had a single variant, 23 (31.9%) had 2 variants and 5 (6.9%) had 3 or more variants (Figure 1, Table 2, Table S2). Among patients without monogenic *HFE* alteration (*n* = 57), 21 (36.8%) had at least one variant identified, with *CP*, *ATP7B*, *SERPINA1* and *GBA* as the most frequently affected genes; single patients carried variants in *ANK1*, *CFTR*, *HBB*, *HNF1B*, *SLC4A1*, *TF*, *TFR2* and *UROD* (Table S3). Notably, variants in *HJV*, *FTL* and *BCS1L* were identified exclusively in patients with concomitant *HFE* variants.

**TABLE 2:** Clinical exome sequencing results in patients with unexplained hyperferritinemia.

Category			No. (%)
Baseline *HFE* genetic testing			
Not performed			67 (62.0)
Negative			19 (17.6)
*HFE* H63D heterozygous			11 (10.2)
*HFE* C282Y heterozygous			6 (5.6)
*HFE* C282Y/H63D (*Cis*/*Trans*)			4 (3.7)
*HFE* H63D homozygous			1 (0.9)
Number of genetic variants identified by the CES			
0			36 (33.3)
1			44 (40.7)
2			23 (21.3)
3			4 (3.7)
4			1 (0.9)
Primary variant (variant 1)			
Variant 1—Gene			
None identified			36 (33.3)
*HFE*			41 (38.0)
*SERPINA1*			6 (5.6)
*ATP7B*			5 (4.6)
*CP*			5 (4.6)
*GBA*			3 (2.8)
*ANK1*			1 (0.9)
*BCS1L*			1 (0.9)
*CFTR*			1 (0.9)
*FTL*			1 (0.9)
*HAMP*			1 (0.9)
*HBB*			1 (0.9)
*HJV*			1 (0.9)
*HNF1B*			1 (0.9)
*SLC4A1*			1 (0.9)
*TF*			1 (0.9)
*TFR2*			1 (0.9)
*UROD*			1 (0.9)
Variant 1—Zygosity			
Heterozygous			66 (91.7)
Homozygous			6 (8.3)
Variant 1—ACMG classification			
VUS			28 (38.9)
Pathogenic			36 (50.0)
Likely pathogenic			8 (11.1)
Variant 1—HGVS Nomenclature c.	AAF^a^	ACMG	
*HFE*: NM_000410.4:c.845G>A (p.Cys282Tyr)	0.057	P	21 (29.2)
*HFE*: NM_000410.4:c.187C>G (p.His63Asp)	0.144	VUS	19 (26.4)
*SERPINA1*: NM_000295.5:c.1096G>A (p.Glu366Lys)	0.018	P	3 (4.2)
*SERPINA1*: NM_000295.5:c.863A>T (p.Glu288Val)	0.037	P	3 (4.2)
*CP*: NM_000096.4:c.2684G>C (p.Gly895Ala)	2.03 × 10^−3^	VUS	2 (2.8)
*GBA*: NM_000157.4:c.1448T>C (p.Leu483Pro)	1.37 × 10^−3^	P	2 (2.8)
*ANK1*: NM_000037.4:c.5504_5507del (p.Ile1835Thrfs*16)	0	P	1 (1.4)
*ATP7B*: NM_000053.4:c.1512dup (p.Asn505*)	0	P	1 (1.4)
*ATP7B*: NM_000053.4:c.230dup (p.Asp78Glyfs*85)	0	P	1 (1.4)
*ATP7B*: NM_000053.4:c.2605G>A (p.Gly869Arg)	1.22 × 10^−3^	LP	1 (1.4)
*ATP7B*: NM_000053.4:c.2978C>T (p.Thr993Met)	1.51 × 10^−4^	LP	1 (1.4)
*ATP7B*: NM_000053.4:c.3275C>T (p.Thr1092Met)	7.90 × 10^−5^	LP	1 (1.4)
*BCS1L*: NM_004328.5:c.460 + 2T>C (Splice Donor)	9.00 × 10^−6^	P	1 (1.4)
*CFTR*: NM_000492.4:c.2991G>C (p.Leu997Phe)	2.60 × 10^−3^	VUS	1 (1.4)
*CP*: NM_000096.4:c.2476_2495del (p.Asn826Leufs*21)	0	LP	1 (1.4)
*CP*: NM_000096.4:c.2520_2523del (p.Thr841Argfs*52)	0	LP	1 (1.4)
*CP*: NM_000096.4:c.2799C>A (p.Asp933Glu)	9.00 × 10^−6^	VUS	1 (1.4)
*FTL*: NM_000146.4:c.89C>T (p.Thr30Ile)	1.80 × 10^−5^	LP	1 (1.4)
*GBA*: NM_000157.4:c.115 + 4A>C (Splice region, near donor)	0	VUS	1 (1.4)
*HAMP*: NM_021175.4:c.212G>A (p.Gly71Asp)	3.02 × 10^−3^	VUS	1 (1.4)
*HBB*: NM_000518.5:c.118C>T (p.Gln40*)	6.77 × 10^−4^	P	1 (1.4)
*HFE*: NM_000410.4:c.854A>C (p.Glu285Ala)	1.80 × 10^−5^	VUS	1 (1.4)
*HJV*: NM_213653.4:c.904G>A (p.Glu302Lys)	3.43 × 10^−4^	VUS	1 (1.4)
*HNF1B* (del 17q12): NC_000017.10:g.(34 892 941_36 104 886)del^b^	0	P	1 (1.4)
*SLC4A1*: NM_000342.4:c.448C>T (p.Arg150*)	0	P	1 (1.4)
*TF*: NM_001063.4:c.1203 + 1G>T (Splice donor)	0	LP	1 (1.4)
*TFR2*: NM_003227.4:c.1118G>A (p.Gly373Asp)	5.65 × 10^−4^	VUS	1 (1.4)
*UROD*: NM_000374.5:c.390G>T (p.Glu130Asp)	2.64 × 10^−4^	LP	1 (1.4)
Secondary variant (variant 2)			
Variant 2—Gene			
None identified			80 (74.1)
*HFE*			13 (12.0)
*SERPINA1*			9 (8.3)
*GBA*			2 (1.9)
*ATP7B*			1 (0.9)
*HAMP*			1 (0.9)
*TF*			1 (0.9)
*TFR2*			1 (0.9)
Variant 2—Zygosity			
Heterozygous			27 (96.4)
Homozygous			1 (3.6)
Variant 2—ACMG Classification			
VUS			18 (64.3)
Pathogenic			9 (32.1)
Likely pathogenic			1 (3.6)
Variant 2—HGVS Nomenclature	AAF^a^	ACMG	
*HFE*: NM_000410.4:c.187C>G (p.His63Asp)	0.144	VUS	12 (42.9)
*SERPINA1*: NM_000295.5:c.1096G>A (p.Glu366Lys)	0.018	P	4 (14.3)
*SERPINA1*: NM_000295.5:c.863A>T (p.Glu288Val)	0.037	P	4 (14.3)
*ATP7B*: NM_000053.4:c.3053C>T (p.Ala1018Val)	4.50 × 10^−5^	P	1 (3.6)
*GBA*: NM_000157.4:c.115 + 3G>A (Splice region, near donor)	0	VUS	1 (3.6)
*GBA*: NM_000157.4:c.247C>T (p.Arg83Cys)	5.30 × 10^−5^	LP	1 (3.6)
*HAMP*: NM_021175.4:c.212G>A (p.Gly71Asp)	3.02 × 10^−3^	VUS	1 (3.6)
*HFE*: NM_000410.4:c.710T>G (p.Met237Arg)	0	VUS	1 (3.6)
*SERPINA1*: NM_000295.5:c.922G>T (p.Ala308Ser)	3.74 × 10^−3^	VUS	1 (3.6)
*TF*: NM_001063.4:c.1925G>A (p.Arg642Gln)	5.30 × 10^−5^	VUS	1 (3.6)
*TFR2*: NM_003227.4:c.1513G>A (p.Val505Met)	4.22 × 10^−4^	VUS	1 (3.6)
Tertiary variant (variant 3)			
Variant 3—Gene			
None identified			103 (95.4)
*HFE*			4 (3.7)
*HBB*			1 (0.9)
Variant 3—Zygosity			
Heterozygous			5 (100.0)
Variant 3—ACMG classification			
VUS			4 (80.0)
Pathogenic			1 (20.0)
Variant 3—HGVS Nomenclature	AAF^a^	ACMG	
*HFE*: NM_000410.4:c.187C>G (p.His63Asp)	0.144	VUS	4 (80.0)
*HBB*: NM_000518.5:c.126_129del (p.Phe42Leufs*19)	0	P	1 (20.0)
Quaternary variant (variant 4)			
Variant 4—Gene			
None identified			107 (99.1)
*HFE*			1 (0.9)
Variant 4—Zygosity			
Heterozygous			1 (100.0)
Variant 4—ACMG Classification			
VUS			1 (100.0)
Variant 4—HGVS Nomenclature	AAF^a^	ACMG	
*HFE*: NM_000410.4:c.187C>G (p.His63Asp)	0.144	VUS	1 (100.0)
Patients with 2 heterozygous variants in the same gene (*Cis*/*Trans*)			10 (9.3)
*HFE*			8 (7.4)
*GBA*			2 (1.9)

*Note:* Data are presented as No. (%) unless otherwise indicated. Percentages are calculated based on the total study population (*N* = 108). HGVS nomenclature follows Human Genome Variation Society guidelines. Gene symbols are presented according to HUGO Gene Nomenclature Committee guidelines.

Abbreviations: AAF, alternative allele frequency; ACMG, American College of Medical Genetics and Genomics; HGVS, Human Genome Variation Society; LP, likely pathogenic; P, pathogenic; VUS, variant of uncertain significance.

^a^ Alternative allele frequencies were obtained from the Genome Aggregation Database (gnomAD) exome dataset, European non‐Finnish population (NFE). Values represent proportions; 0 indicates variant not observed in the reference population.

^b^ The *HNF1B* variant is a 1.21 Mb deletion at 17q12 (seq[GRCh37] del(17)(q12); chr17:34892941–36 104 886; NC_000017.10:g.(34 892 941_36 104 886)del) encompassing 17 loci: *HNF1B*, *PIGW*, *MRM1*, *DHRS11*, *LHX1*, *C17orf78*, *DDX52*, *ACACA*, *SNORA90*, *GGNBP2*, *TADA2A*, *SYNRG*, *AATF*, *MIR378J*, *DUSP14*, *LHX1‐DT* and *MIR2909*. The deletion was confirmed using chromosomal microarray analysis in an independent ISO 15189‐accredited laboratory. The phenotypic effect was attributed to *HNF1B* haploinsufficiency based on ClinGen Haploinsufficiency score of 3, dominant inheritance pattern associated with hepatic manifestations (OMIM #614527, GeneReviews NBK401562, and reference [18]), and high probability of loss‐of‐function intolerance (pLI = 1; o/e LOF upper = 0.17; DECIPHER HI score = 0.76). Other genes in the region have no established disease association, follow autosomal recessive inheritance (*PIGW*, *ACACA*) or lack evidence of haploinsufficiency.

Regarding the number of genes affected per patient, among the 72 patients with at least one variant (VUS, LP, or P), 50 (69.4%) exhibited monogenic involvement with variants in a single gene, whereas 22 patients (30.6%) demonstrated digenic or oligogenic patterns with variants in multiple genes. When restricted to LP/P variants (*n* = 44), 35 patients (79.5%) showed monogenic involvement, whereas nine patients (20.5%) exhibited digenic or oligogenic architecture (Figure 1, Table S4).

**FIGURE 2:**
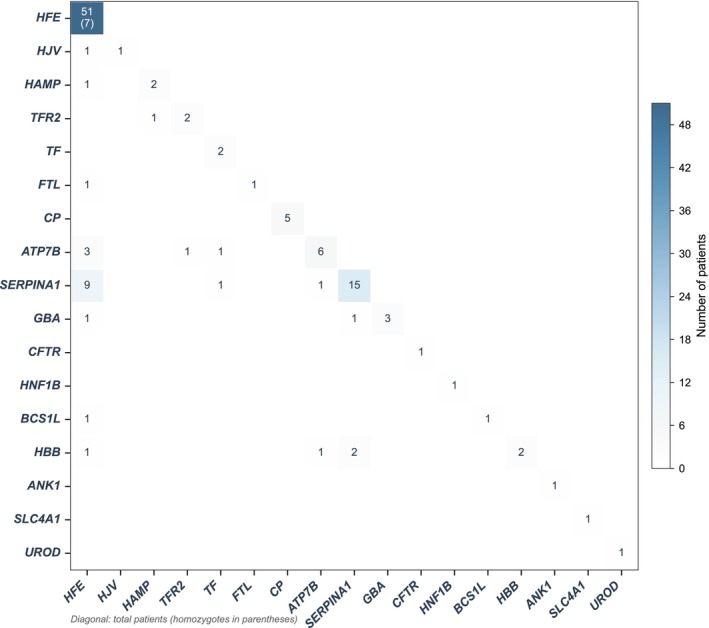
Gene co‐occurrence heatmap including all retained variants. Heatmap displaying gene co‐occurrence patterns among patients carrying VUS, LP or P variants. Diagonal values indicate the total number of patients with variants in each gene (patients with two variants in the same gene are counted once), with homozygous patients shown in parentheses. Off‐diagonal values represent the number of patients harbouring variants in both genes, indicating potential digenic involvement. *HFE* was involved in 51 patients (7 homozygous), followed by *SERPINA1* (*n* = 15), *ATP7B* (*n* = 6), *CP* (*n* = 5), *GBA* (*n* = 3), *TF* (*n* = 2), *TFR2* (*n* = 2), *HAMP* (*n* = 2) and *HBB* (*n* = 2). The most frequent digenic combinations involved *HFE* and *SERPINA1* (*n* = 9), *HFE* and *ATP7B* (*n* = 3), and *SERPINA1* and *HBB* (*n* = 2). Gene symbols are presented according to HUGO Gene Nomenclature Committee guidelines.

Variants (VUS, LP, or P) were identified across 17 genes in the study population (Table S5). *HFE* was the most frequently affected gene (51 patients [47.2%]), followed by *SERPINA1* (15 [13.9%]), *ATP7B* (6 [5.6%]), *CP* (5 [4.6%]) and *GBA* (3 [2.8%]). These genes mapped to four functional pathways: IRON‐SENS (56 patients), IRON‐TRANS (14 patients), HEPATO‐METAB (21 patients), and ERYTHRO‐HEME (5 patients), with some patients contributing to multiple pathways. Gene co‐occurrence analysis among patients with VUS, LP or P variants identified *HFE* and *SERPINA1* as the most frequent digenic combination (*n* = 9), followed by *HFE* and *ATP7B* (*n* = 3), and *SERPINA1* and *HBB* (*n* = 2) (Figure 2, Table S6). When restricted to LP/P variants, 13 genes carried variants; *HFE* remained the most frequently affected (21 patients [19.4%]), followed by *SERPINA1* (14 [13.0%]), *ATP7B* (6 [5.6%]) and *CP* (2 [1.9%]) (Table S7). The most frequent LP/P digenic combination was *HFE* and *SERPINA1* (*n* = 4), followed by *SERPINA1* and *HBB* (*n* = 2) (Figure 2, Figure S1, Table S8). Variant‐level distribution and co‐occurrence patterns are reported in Tables S9–S12.

### 3.3 Functional Pathway Distribution

Among patients with at least one retained variant (VUS, LP or P) (*n* = 72), 55 (76.4%) had variants mapping exclusively to a single functional pathway: 38 (52.8%) in IRON‐SENS, 7 (9.7%) in IRON‐TRANS, 7 (9.7%) in HEPATO‐METAB, and 3 (4.2%) in ERYTHRO‐HEME. Seventeen patients (23.6%) carried variants mapping to multiple pathways, representing 77.3% of the 22 patients with digenic or oligogenic inheritance; the most common cross‐pathway combinations were IRON‐SENS with HEPATO‐METAB (10 [13.9%]) or IRON‐TRANS (4 [5.6%]) (Table S13, Figures S2 and S3). When restricted to LP/P variants (*n* = 44), 36 patients (81.8%) had variants in a single pathway: 16 (36.4%) in IRON‐SENS, 10 (22.7%) in HEPATO‐METAB, 7 (15.9%) in IRON‐TRANS and 3 (6.8%) in ERYTHRO‐HEME. Eight patients (18.2%) carried LP/P variants mapping to multiple pathways, most commonly IRON‐SENS combined with HEPATO‐METAB (4 [9.1%]) (Figures S4 and S5).

### 3.4 Genotype–Phenotype Correlations

**FIGURE 3:**
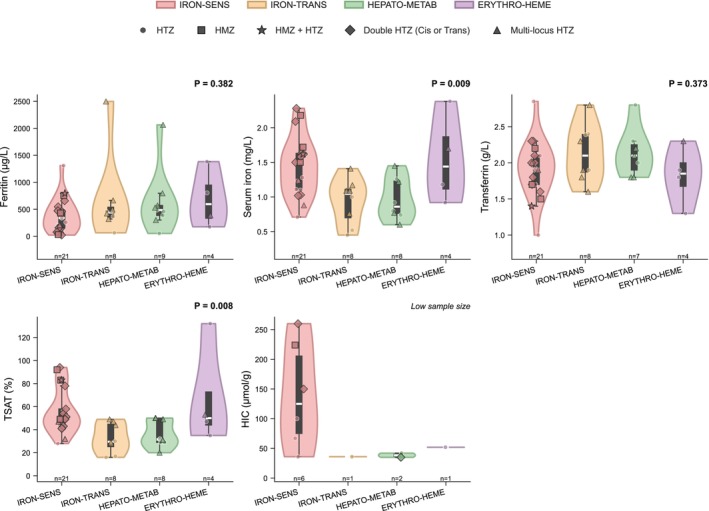
Iron metabolism parameters by functional pathway in patients with likely pathogenic or pathogenic variants. Violin plots showing the distribution of iron metabolism parameters across four functional pathway groups in patients with LP or P variants only. Each plot displays the median (white line), interquartile range (black box) and individual data points stratified by zygosity: Heterozygous (HTZ, circles), homozygous (HMZ, squares), homozygous with additional heterozygous variant (HMZ + HTZ, stars), double heterozygous in the same gene in *cis* or *trans* configuration (diamonds), and multi‐locus heterozygous (triangles). Sample sizes are indicated below each group and may vary across parameters owing to missing data. *p*‐values are from Kruskal–Wallis tests. Serum ferritin concentrations did not differ significantly across pathway groups (*p* = 0.382). Serum iron was significantly higher in the IRON‐SENS group compared with other pathway groups (*p* = 0.009). Transferrin saturation was significantly elevated in the IRON‐SENS group (*p* = 0.008). The five patients with the highest transferrin saturation values in the IRON‐SENS group all harboured *HFE* variants: Two were homozygous for p.Cys282Tyr, one was homozygous for p.His63Asp, and two were compound heterozygous for p.Cys282Tyr and p.His63Asp, including one patient with a concomitant *SERPINA1* p.Glu366Lys variant. Statistical comparison of hepatic iron concentration was not performed owing to sparse data precluding valid inference (IRON‐SENS, *n* = 6; IRON‐TRANS, *n* = 1; HEPATO‐METAB, *n* = 2; ERYTHRO‐HEME, *n* = 1); however, patients in the IRON‐SENS group exhibited hepatic iron concentrations consistently above the pathological threshold of 36 μmol/g.

Iron metabolism parameters were compared across the four functional pathway groups among patients with LP/P variants (Table 3, Figure 3). Serum iron was significantly higher in the IRON‐SENS group (median, 1.5 mg/L [IQR, 1.1–1.6]) compared with the IRON‐TRANS (1.0 mg/L [IQR, 0.7–1.1]) and HEPATO‐METAB (0.9 mg/L [IQR, 0.8–1.2]) groups (*p* = 0.009). Similarly, transferrin saturation was significantly elevated in the IRON‐SENS group (median, 49% [IQR, 43%–58%]) compared with the IRON‐TRANS (29.5% [IQR, 25.2%–44.8%]) and HEPATO‐METAB (31.5% [IQR, 29.0%–49.2%]) groups (*p* = 0.008). These differences were not maintained when including all retained variants (VUS, LP and P) (Table 3, Figure S6). Statistical comparison of hepatic iron concentration was not performed owing to sparse data. Patients in the IRON‐SENS group exhibited hepatic iron concentrations consistently above the pathological threshold of 36 μmol/g (median, 125.0 μmol/g [IQR, 75.2–205.5]). Iron metabolism parameters were also compared across the most frequent digenic combinations and monogenic *HFE* genotypes (Figure S7). Although limited by small sample sizes, patients with *HFE*/*SERPINA1* and *HFE*/*ATP7B* combinations exhibited transferrin saturation below 50%, whereas patients with homozygous *HFE* variants or double heterozygous *HFE* variants frequently exceeded this threshold. Regarding *ATP7B*, six patients each carried a single heterozygous variant, including three pathogenic and three likely pathogenic variants. Ceruloplasmin concentrations were slightly below or at the lower limit of normal in all cases (reference range, 0.20–0.60 g/L), with values below the lower limit in patients with pathogenic variants (range, 0.17–0.19 g/L) and at the lower limit of normal in patients with likely pathogenic variants (range, 0.22–0.28 g/L).

**TABLE 3:** Comparison of iron metabolism and liver parameters across functional gene pathway categories.

Variable	IRON‐SENS	IRON‐TRANS	HEPATO‐METAB	ERYTHRO‐HEME	*p* ^a^	*η* ^2^
	(*n* = 22)	(*n* = 8)	(*n* = 10)	(*n* = 4)		
A. Likely pathogenic/pathogenic variants						
Serum ferritin, μg/L [M: 22–322, F: 10–291]^b^	340.0 [158.0–478.6]	439.2 [354.5–537.5]	486.0 [386.0–571.0]	595.0 [334.2–947.8]	0.382	0.002
Serum iron, mg/L [M: 0.65–1.75, F: 0.5–1.7]^b^	1.5 [1.1–1.6]	1.0 [0.7–1.1]	0.9 [0.8–1.2]	1.4 [1.1–1.9]	0.009	0.230
Transferrin, g/L [M: 2.15–3.65 F: 2.5–3.8]^b^	1.9 [1.7–2.1]	2.1 [1.9–2.4]	2.1 [1.9–2.2]	1.9 [1.7–2.0]	0.373	0.003
Transferrin saturation, % [20–40]^b^	49.0 [43.0–58.0]	29.5 [25.2–44.8]	31.5 [29.0–49.2]	50.0 [44.0–72.8]	0.008	0.242
Hepatic iron concentration, μmol/g [5–27]	125.0 [75.2–205.5]	36.0 [36.0–36.0]	38.5 [36.8–40.2]	52.0 [52.0–52.0]	*Low n*	—
Liver stiffness, kPa [< 5]	4.8 [4.3–5.7]	4.7 [4.2–5.6]	6.0 [5.2–6.5]	21.0 [21.0–21.0]	0.258	0.049
ASAT, U/L [20–40]^b^	25.0 [19.0–31.0]	24.0 [20.8–25.8]	27.5 [21.2–35.2]	29.0 [25.8–32.5]	0.788	0.000
ALAT, U/L [7–40]^b^	25.0 [19.0–40.0]	28.5 [21.8–48.2]	26.5 [16.8–42.8]	17.5 [14.0–27.5]	0.597	0.000
GGT, U/L [M: ≤ 73, F: ≤ 38]^b^	31.0 [17.0–44.0]	27.0 [18.0–42.2]	35.5 [18.2–52.5]	65.0 [44.5–128.0]	0.644	0.000

Variable	IRON‐SENS	IRON‐TRANS	HEPATO‐METAB	ERYTHRO‐HEME	*p* ^a^	*η* ^2^
	(*n* = 45)	(*n* = 11)	(*n* = 12)	(*n* = 4)		
B. All retained variants						
Serum ferritin, μg/L [M: 22–322, F: 10–291]^b^	475.8 [199.2–686.0]	423.0 [331.0–580.0]	508.0 [404.2–740.5]	595.0 [334.2–947.8]	0.829	0.000
Serum iron, mg/L [M: 0.65–1.75, F: 0.5–1.7]^b^	1.2 [1.0–1.6]	1.1 [0.9–1.4]	0.9 [0.8–1.3]	1.4 [1.1–1.9]	0.289	0.013
Transferrin, g/L [M: 2.15–3.65 F: 2.5–3.8]^b^	2.0 [1.7–2.2]	2.0 [1.9–2.4]	2.2 [1.9–2.3]	1.9 [1.7–2.0]	0.267	0.016
Transferrin saturation, % [20–40]^b^	47.0 [37.5–54.5]	44.0 [28.5–52.0]	32.0 [29.0–50.0]	50.0 [44.0–72.8]	0.288	0.013
Hepatic iron concentration, μmol/g [5–27]	67.0 [42.5–125.0]	36.0 [36.0–43.0]	38.0 [36.5–40.0]	52.0 [52.0–52.0]	*Low n*	—
Liver stiffness, kPa [< 5]	5.1 [3.7–6.4]	4.9 [4.4–5.8]	6.0 [4.8–6.8]	21.0 [21.0–21.0]	0.273	0.021
ASAT, U/L [20–40]^b^	26.0 [21.0–40.0]	25.0 [22.0–27.5]	29.0 [23.0–45.0]	29.0 [25.8–32.5]	0.901	0.000
ALAT, U/L [7–40]^b^	30.0 [20.5–50.0]	32.0 [24.0–55.0]	31.0 [17.0–51.0]	17.5 [14.0–27.5]	0.444	0.000
GGT, U/L [M: ≤ 73, F: ≤ 38]^b^	31.5 [19.2–68.5]	26.0 [21.5–43.5]	36.0 [21.0–90.0]	65.0 [44.5–128.0]	0.780	0.000

*Note:* Data are presented as median [interquartile range]. *η* ^2^ (eta‐squared) effect size interpretation: ≥ 0.01, small effect; ≥ 0.06, medium effect; ≥ 0.14, large effect (Cohen conventions). IRON‐SENS, systemic iron sensing and hepcidin regulation (*HFE*, *HJV*, *HAMP*, *TFR2*); IRON‐TRANS, iron transport and storage (*TF*, *CP*, *FTL*, *ATP7B*); HEPATO‐METAB, hepatic metabolism and lysosomal storage (*SERPINA1*, *HNF1B*, *GBA*, *CFTR*, *BCS1L*); ERYTHRO‐HEME, erythropoiesis and heme biosynthesis (*HBB*, *ANK1*, *UROD*, *SLC4A1*).

Abbreviations: ALAT, alanine aminotransferase; ASAT, aspartate aminotransferase; GGT, gamma‐glutamyltransferase.

^a^ Kruskal–Wallis test.

^b^ The reference values are adapted to an adult population.

## 4 Discussion

In this study, we characterised the genetic landscape of 108 patients with hyperferritinemia who underwent CES at a tertiary referral center. CES identified at least one LP or P variant in 40.7% of patients. Digenic or oligogenic inheritance was observed in approximately one‐third of patients harbouring at least one variant and one‐fifth of those with LP/P variants, most frequently *HFE*‐*SERPINA1* and *HFE*‐*ATP7B*. Cross‐pathway combinations occurred in more than three‐quarters of digenic patients, predominantly IRON‐SENS plus HEPATO‐METAB and IRON‐SENS plus IRON‐TRANS, although the small sample sizes within certain pathway groups, particularly ERYTHRO‐HEME, warrant cautious interpretation. Despite these limitations, these findings suggest genetic heterogeneity underlying hyperferritinemia and support the potential utility of comprehensive genomic testing in this population.

Our findings can be contextualised with previous studies in atypical iron disorders and iron overload. McDonald et al. reported a definitive molecular diagnosis in five of eight patients with atypical iron disorders using a targeted 39‐gene panel, although this high yield likely reflects the highly selected nature of their cohort [14]. Larger studies have generally reported lower yields. Lanktree et al. identified non‐*HFE* hemochromatosis in only 3.2% of 190 patients with suspected iron overload [12]. Viveiros et al. using magnetic resonance imaging‐based patient selection, found likely pathogenic variants in 38% of 180 patients with confirmed hepatic iron overload [19]. Interestingly, among patients without monogenic *HFE* alteration, more than one‐third had at least one variant identified, and *CP* ranked among the top affected genes. These findings align with a targeted sequencing study of 32 iron‐related genes in patients with nonalcoholic fatty liver disease, which demonstrated that *CP* variants were independently associated with hyperferritinemia, hepatic siderosis, and more severe liver fibrosis [20]. The identification of *CP* variants in our cohort supports the relevance of this gene in the genetic architecture of hyperferritinemia beyond classic hemochromatosis genes.

Several factors may account for the relatively high variant detection rate in this study. First, patients were systematically referred to a tertiary center after exclusion of secondary causes of hyperferritinemia by their treating physicians, representing a population with higher pretest probability of a genetic aetiology. Second, our use of CES rather than targeted gene panels allowed identification of variants in genes beyond those traditionally associated with hemochromatosis, including *SERPINA1*, *GBA* and *ATP7B*.

A notable finding of this study is the substantial proportion of patients exhibiting digenic or oligogenic architecture. These observations support the emerging concept that hyperferritinemia and iron overload disorders frequently involve complex inheritance patterns rather than simple monogenic Mendelian inheritance. The concept of digenic inheritance in iron metabolism disorders is not new. Merryweather‐Clarke et al. described digenic inheritance involving mutations in *HAMP* and *HFE* that resulted in different hemochromatosis phenotypes [21]. Similarly, Jacolot et al. and Le Gac et al. demonstrated that variants in *HAMP* and *HJV*, respectively, can modify the phenotypic expression of *HFE*:p.C282Y homozygosity [22, 23]. Our findings extend these observations by demonstrating that multilocus involvement may be more common than previously appreciated, particularly when variants across multiple functional pathways are considered.

The most frequent digenic combinations in the present study involved *HFE* and *SERPINA1*, reflecting the high carrier frequency of both *HFE* variants in European Non‐Finnish populations (approximately 6% for p.C282Y and 14% for p.H63D heterozygosity) and *SERPINA1* variants (approximately 2% for the PI*Z allele). Whether such combinations represent true digenic inheritance with synergistic effects on iron metabolism or coincidental co‐occurrence requires further functional investigation. In a genome‐wide association study conducted in the UK Biobank with a replication in a hospital‐based cohort from the Michigan Genomics Initiative, *HFE* p.C282Y and *SERPINA1* p.E366K (PI*Z) were among the top variants associated with cirrhosis [24]. These observations support the biological plausibility of epistatic interactions between *HFE* and *SERPINA1* variants in the pathogenesis of chronic liver disease [25]. Notably, a recent exome‐based genotype‐first reverse phenotyping study demonstrated that nonbenign *SERPINA1* variants were associated with liver manifestations, including hyperferritinemia in 23% of carriers, through a dominant effect [9]. These findings corroborate the clinical relevance of the *SERPINA1* PI*Z variant identified in our study [9]. Regarding the *SERPINA1* PI*S variant, this allele has been associated with hyperferritinemia in patients with metabolic dysfunction‐associated steatotic liver disease, with median serum ferritin of 554 (298–1003) μg/L in PI*S heterozygotes compared with 348 (201–468) μg/L in patients without PI*Z or PI*S variants, independently of *HFE* genotypes [26]. However, evidence supporting a direct role of the PI*S variant in hyperferritinemia remains less robust than that for PI*Z. Regarding the estimation of co‐occurrence probability for polymorphic variants in *HFE* and *SERPINA1*, based on Hardy–Weinberg equilibrium and observed allele frequencies in the gnomAD exome dataset, the expected frequency for an individual to carry heterozygous variants in both genes in the European Non‐Finnish population ranges from 1.76% (1 in 57) for *HFE* p.His63Asp with *SERPINA1* PI*S to 0.38% (1 in 263) for *HFE* p.Cys282Tyr with *SERPINA1* PI*Z (Table S10). Despite the polymorphic nature of these variants, the probability of co‐occurrence remains low in the general population.

To provide a clinically meaningful framework for interpreting the genetic heterogeneity observed, we classified variants into four functional pathway groups based on their established roles in iron homeostasis. This classification aligns with the pathophysiological mechanisms through which different genetic defects lead to hyperferritinemia and may have implications for clinical management. Patients with LP or P variants in the IRON‐SENS pathway exhibited significantly higher transferrin saturation and serum iron concentrations compared with patients harbouring variants in other pathways. This biochemical signature reflects the underlying pathophysiology of hemochromatosis, in which impaired hepcidin regulation leads to unrestricted iron absorption with elevated circulating iron. Although hepatic iron concentration data were limited, patients in the IRON‐SENS group showed the highest values, consistent with parenchymal iron loading characteristic of hereditary hemochromatosis [2, 4]. In contrast, patients with variants in the HEPATO‐METAB pathway (including *SERPINA1*, *GBA* and related genes) exhibited lower transferrin saturation despite elevated ferritin, consistent with secondary hyperferritinemia resulting from hepatocellular dysfunction or inflammation rather than primary iron overload. The observation that iron metabolism parameters differed significantly across functional pathway groups only among patients with LP or P variants, whereas these differences were not maintained when VUS were included, supports the clinical relevance of stringent variant classification. This pattern suggests that LP and P variants exert phenotypic effects on iron homeostasis with sufficient magnitude to be detected despite limited statistical power, whereas VUS may represent variants with smaller or no functional impact.

Our findings have several implications for the diagnostic evaluation of patients with hyperferritinemia. First, the high rate of variant detection supports the utility of comprehensive genomic testing in patients with unexplained hyperferritinemia after exclusion of secondary causes. The 2022 EASL Clinical Practice Guidelines on haemochromatosis recommend that patients who are not homozygous for *HFE*:p.C282Y undergo testing for rare variants to provide a definite diagnosis, improve disease prognostication, and enable family screening [4]. Our data support this recommendation and suggest that CES may be a reasonable first‐tier approach in selected patients, particularly when the clinical presentation is atypical or when targeted panel testing has been uninformative. Furthermore, the findings from the present study support performing targeted *HFE* genotyping before CES in patients with hyperferritinemia as it enables identification of the most common cause of hereditary hemochromatosis at lower cost and shorter turnaround time, reserving clinical exome sequencing for patients without *HFE* p.Cys282Tyr homozygosity. Second, the identification of variants across multiple functional pathways emphasises the importance of considering differential diagnoses beyond hereditary hemochromatosis. The finding of *SERPINA1* variants in 14% of patients underscores the potential for α_1_‐antitrypsin deficiency to present with hyperferritinemia, often in the absence of classic pulmonary manifestations. Similarly, variants in *ATP7B* and *GBA* highlight the phenotypic overlap between primary iron disorders and other inherited metabolic diseases. However, a cautious interpretation is warranted as these genes are associated with autosomal recessive diseases, and monoallelic variants are not established causes of hyperferritinemia. Indirect mechanisms may nonetheless exist. ATP7B is required for copper delivery to ceruloplasmin and reduced ATP7B function leads to decreased ceruloplasmin levels. Ceruloplasmin is a ferroxidase that oxidises Fe^2+^ to Fe^3+^ for iron loading onto transferrin and iron export from hepatocytes and macrophages. Impaired ceruloplasmin ferroxidase activity would therefore lead to intracellular iron accumulation, which stimulates ferritin synthesis as a protective mechanism against iron toxicity [27, 28, 29, 30]. Iron overload has been documented in Wilson disease [31, 32, 33], and ceruloplasmin gene variants have been associated with hyperferritinemia in patients with non‐alcoholic fatty liver disease [20]. Regarding *GBA*, hyperferritinemia is a frequent finding in type 1 Gaucher disease, resulting from iron sequestration in Gaucher cells without elevation of transferrin saturation [34, 35]. This biochemical profile aligns with the hyperferritinemia without iron overload phenotype. As Gaucher disease represents the most common lysosomal storage disorder and isolated hyperferritinemia may be the presenting sign [36], its identification enables access to enzyme replacement therapy, which normalises ferritin levels [37, 38]. Whether monoallelic variants in *ATP7B* and *GBA* exert sufficient functional impact warrants further investigation. Third, the significant differences in iron metabolism parameters across pathway groups may aid in the interpretation of genetic findings. A pattern of elevated transferrin saturation with elevated ferritin suggests involvement of the IRON‐SENS pathway and true hemochromatosis, whereas isolated hyperferritinemia with normal or low transferrin saturation should prompt consideration of alternative etiologies.

Several limitations merit consideration. The retrospective design and single‐center setting may limit generalisability, with potential selection bias towards more severe or atypical presentations. The functional significance of many variants, particularly VUS, remains uncertain and requires careful clinical interpretation. Hepatic iron concentration data were available for only a subset of patients, precluding robust genotype–phenotype correlations with this phenotypic marker. Finally, the sample size limited statistical power for subgroup analyses within individual pathway groups. The identification of variants of interest in 17 genes is consistent with the relatively limited number of genes biologically relevant to iron metabolism phenotypes. Although the CES panel covers approximately 6700 genes, only 251 are directly associated with iron metabolism or hyperferritinemia based on curated disease‐specific panels and Human Phenotype Ontology annotations. A larger cohort might have allowed the identification of associations in genes not detected in the present study, underscoring the value of CES performed in accordance with ISO 15189 accreditation in a routine setting, which enables comprehensive genetic evaluation and reanalysis over time.

CES identified clinically relevant variants in a substantial proportion of patients with unexplained hyperferritinemia, revealing genetic heterogeneity beyond the traditional Mendelian framework, with frequent non‐*HFE* gene involvement and digenic inheritance in approximately one‐third of cases. While current guidelines recommend testing for rare hemochromatosis genes without consensus on comprehensive genomic approaches, our findings suggest that CES should be considered after exclusion of *HFE* p.Cys282Tyr homozygosity.

## Author Contributions

**Paul Morel:** formal analysis, investigation, data curation, writing of the original draft, and review and editing of the manuscript. **Maël Silva Rodriguez:** formal analysis, investigation, data curation, writing of the original draft, and review and editing. **Cyriaque Benmouffek:** formal analysis, investigation, data curation, writing of the original draft, and review and editing. **Betul Goksen:** software development and review and editing. **Céline Chéry:** software development and review and editing. **Mouni Bensenane:** resources and review and editing. **Vincent Haghnejad:** resources and review and editing. **François Feillet:** resources and review and editing. **Jean‐Louis Guéant:** resources and review and editing. **Farès Namour:** methodology, validation, resources, and review and editing. **Jean‐Pierre Bronowicki:** resources and review and editing. **Abderrahim Oussalah:** conceptualisation, methodology, software development, validation, formal analysis, investigation, resources, writing of the original draft, review and editing, visualisation, supervision, project administration, and funding acquisition. All authors approved the final draft submitted.

## Funding

This study was supported by the Department of Genomic Medicine, Division of Biochemistry, Molecular Biology, and Nutrition, University Hospital of Nancy, F‐54000 Nancy, France; and the GenomeDx program, ORION, Université d'Excellence, Université de Lorraine (France). The sponsors had no role in the study design; in the collection, analysis, and interpretation of data; in the writing of the report; or in the decision to submit the manuscript for publication.

## Ethics Statement

The Ethics Committee of the University Hospital of Nancy approved the study protocol (ID: 2020/264). The Nancy Biochemical Database is registered with the French National Commission on Informatics and Liberty (CNIL No. 1763197v0). Written informed consent was obtained from all patients prior to inclusion.

## Conflicts of Interest

Abderrahim Oussalah reports consulting fees from Mirum, Dexter, and Atheneum Partners; lecture fees from Bayer Healthcare and Mirum; travel support from Bayer Healthcare, Gilead Sciences, Mirum, AstraZeneca, Pfizer, and Thermo Fisher Diagnostics; and two pending patents: DNA Methylation Signature for Diagnosing Hepatocellular Carcinoma (EP21306459) and Computer‐Implemented Method for Quality Control Processing of High‐Throughput Sequenced Genomic Data (EP24306620), filed with INSERM Transfert, Satt, Incubateur Lorrain, and CHRU Nancy. Jean‐Pierre Bronowicki reports grants from Roche, AstraZeneca, and MSD (payments to institution); consulting fees from Roche, AstraZeneca, and Bayer; lecture fees from Roche, AstraZeneca, and Bayer; and travel support from Roche and AstraZeneca. Vincent Haghnejad reports a fellowship grant from the French Association for the Study of the Liver (AFEF); lecture fees from Gilead, Ipsen, and Etypharm; and travel support from AbbVie, Ipsen, Gilead, and Roche. Paul Morel, Maël Silva Rodriguez, Cyriaque Benmouffek, Betul Goksen, Céline Chéry, Mouni Bensenane, François Feillet, Jean‐Louis Guéant, and Farès Namour declares no conflicts of interest.

## Supporting information


**Table S1:** List of genes included in variant filtering by panel and Human Phenotype Ontology annotation.
**Table S2:** Clinical exome sequencing results restricted to likely pathogenic and pathogenic variants (*N* = 108).
**Table S3:** Clinical exome sequencing results in patients without monogenic *HFE* disease (*N* = 57).
**Table S4:** Genetic architecture according to variant classification (*N* = 108).
**Table S5:** Gene‐level variant distribution across the study population (VUS, likely pathogenic and pathogenic; *N* = 108).
**Table S6:** Gene co‐occurrence patterns suggestive of digenic or oligogenic inheritance (VUS, likely pathogenic and pathogenic).
**Table S7:** Gene‐level variant distribution restricted to likely pathogenic and pathogenic variants (*N* = 108).
**Table S8:** Gene co‐occurrence patterns restricted to likely pathogenic and pathogenic variants.
**Table S9:** Variant‐level summary across the study population (VUS, likely pathogenic and pathogenic; *N* = 108)
**Table S10:** Variant co‐occurrence patterns (VUS, likely pathogenic and pathogenic).
**Table S11:** Variant‐level summary restricted to likely pathogenic and pathogenic variants (*N* = 108).
**Table S12:** Variant co‐occurrence patterns restricted to likely pathogenic and pathogenic variants.
**Table S13:** Patient classification by functional gene pathway.
**Figure S1:** Gene co‐occurrence heatmap restricted to likely pathogenic and pathogenic variants.
**Figure S2:** Functional pathway distribution including all retained variants.
**Figure S3:** Pairwise pathway overlaps including all retained variants.
**Figure S4:** Functional pathway distribution restricted to likely pathogenic and pathogenic variants.
**Figure S5:** Pairwise pathway overlaps restricted to likely pathogenic and pathogenic variants.
**Figure S6:** Iron metabolism parameters by functional pathway including all retained variants.
**Figure S7:** Iron metabolism parameters in patients with digenic *HFE*/*SERPINA1*, *HFE*/*ATP7B* and *SERPINA1*/*HBB* combinations compared with monogenic *HFE* genotypes.

## Data Availability

Under French law, genetic data are subject to specific regulatory restrictions that preclude their sharing outside the institution where they were generated. Anonymised clinical and biochemical data from this study may be available to qualified researchers upon reasonable request. Requests will be reviewed by the Institutional Review Board of the University Hospital of Nancy and require submission of a research proposal, a statistical analysis plan, and execution of a data‐sharing agreement.

## References

[1] P. C. Adams , D. M. Reboussin , J. C. Barton , et al., “Hemochromatosis and Iron‐Overload Screening in a Racially Diverse Population,” New England Journal of Medicine 352, no. 17 (2005): 1769–1778. 15858186 10.1056/NEJMoa041534

[2] P. C. Adams and J. C. Barton , “A Diagnostic Approach to Hyperferritinemia With a Non‐Elevated Transferrin Saturation,” Journal of Hepatology 55, no. 2 (2011): 453–458. 21354228 10.1016/j.jhep.2011.02.010

[3] J. O. Cullis , E. J. Fitzsimons , W. J. Griffiths , E. Tsochatzis , and D. W. Thomas , “Investigation and Management of a Raised Serum Ferritin,” British Journal of Haematology 181, no. 3 (2018): 331–340. 29672840 10.1111/bjh.15166

[4] European Association for the Study of the Liver , “EASL Clinical Practice Guidelines on Haemochromatosis,” Journal of Hepatology 77, no. 2 (2022): 479–502. 35662478 10.1016/j.jhep.2022.03.033

[5] K. V. Kowdley , K. E. Brown , J. Ahn , and V. Sundaram , “ACG Clinical Guideline: Hereditary Hemochromatosis,” American Journal of Gastroenterology 114, no. 8 (2019): 1202–1218. 31335359 10.14309/ajg.0000000000000315

[6] A. Pietrangelo , “Hereditary Hemochromatosis: Pathogenesis, Diagnosis, and Treatment,” Gastroenterology 139, no. 2 (2010): 393–408. 20542038 10.1053/j.gastro.2010.06.013

[7] L. G. Biesecker and R. C. Green , “Diagnostic Clinical Genome and Exome Sequencing,” New England Journal of Medicine 370, no. 25 (2014): 2418–2425. 24941179 10.1056/NEJMra1312543

[8] T. Alix , C. Chery , T. Josse , et al., “Predictors of the Utility of Clinical Exome Sequencing as a First‐Tier Genetic Test in Patients With Mendelian Phenotypes: Results From a Referral Center Study on 603 Consecutive Cases,” Human Genomics 17, no. 1 (2023): 5. 36740706 10.1186/s40246-023-00455-x PMC9899384

[9] M. Silva Rodriguez , M. Mulot , C. Chery , et al., “Exome‐Based Genotype‐First Reverse Phenotyping Using Structured Electronic Health Record Data Identifies Novel SERPINA1 Variants Associated With Liver Markers and Demonstrates a Dominant Effect for Specific Variants on Liver Phenotype,” Hepatology Research 55, no. 7 (2025): 1075–1092. 40359317 10.1111/hepr.14203

[10] S. Badar , F. Busti , A. Ferrarini , et al., “Identification of Novel Mutations in Hemochromatosis Genes by Targeted Next Generation Sequencing in Italian Patients With Unexplained Iron Overload,” American Journal of Hematology 91, no. 4 (2016): 420–425. 26799139 10.1002/ajh.24304

[11] A. Del‐Castillo‐Rueda , M. I. Moreno‐Carralero , N. Cuadrado‐Grande , et al., “Mutations in the HFE, TFR2, and SLC40A1 Genes in Patients With Hemochromatosis,” Gene 508, no. 1 (2012): 15–20. 22890139 10.1016/j.gene.2012.07.069

[12] M. B. Lanktree , B. Sadikovic , J. S. Waye , et al., “Clinical Evaluation of a Hemochromatosis Next‐Generation Sequencing Gene Panel,” European Journal of Haematology 98, no. 3 (2017): 228–234. 27753142 10.1111/ejh.12820

[13] G. Le Gac , C. Ka , R. Joubrel , et al., “Structure‐Function Analysis of the Human Ferroportin Iron Exporter (SLC40A1): Effect of Hemochromatosis Type 4 Disease Mutations and Identification of Critical Residues,” Human Mutation 34, no. 10 (2013): 1371–1380. 23784628 10.1002/humu.22369

[14] C. J. McDonald , L. Ostini , D. F. Wallace , A. Lyons , D. H. Crawford , and V. N. Subramaniam , “Next‐Generation Sequencing: Application of a Novel Platform to Analyze Atypical Iron Disorders,” Journal of Hepatology 63, no. 5 (2015): 1288–1293. 26151776 10.1016/j.jhep.2015.06.027

[15] A. Piperno , S. Pelucchi , and R. Mariani , “Inherited Iron Overload Disorders,” Translational Gastroenterology and Hepatology 5 (2020): 25. 32258529 10.21037/tgh.2019.11.15 PMC7063521

[16] G. Ravasi , S. Pelucchi , F. Bertola , M. M. Capelletti , R. Mariani , and A. Piperno , “Identification of Novel Mutations by Targeted NGS Panel in Patients With Hyperferritinemia,” Genes (Basel) 12, no. 11 (2021): 1778. 34828384 10.3390/genes12111778 PMC8623017

[17] S. Pelucchi , R. Mariani , A. Salvioni , et al., “Novel Mutations of the Ferroportin Gene (SLC40A1): Analysis of 56 Consecutive Patients With Unexplained Iron Overload,” Clinical Genetics 73, no. 2 (2008): 171–178. 18177470 10.1111/j.1399-0004.2007.00950.x

[18] D. Dubois‐Laforgue , E. Cornu , C. Saint‐Martin , et al., “Diabetes, Associated Clinical Spectrum, Long‐Term Prognosis, and Genotype/Phenotype Correlations in 201 Adult Patients With Hepatocyte Nuclear Factor 1B (HNF1B) Molecular Defects,” Diabetes Care 40, no. 11 (2017): 1436–1443. 28420700 10.2337/dc16-2462

[19] A. Viveiros , B. Schaefer , M. Panzer , et al., “MRI‐Based Iron Phenotyping and Patient Selection for Next‐Generation Sequencing of Non‐Homeostatic Iron Regulator Hemochromatosis Genes,” Hepatology 74, no. 5 (2021): 2424–2435. 34048062 10.1002/hep.31982 PMC8596846

[20] E. Corradini , E. Buzzetti , P. Dongiovanni , et al., “Ceruloplasmin Gene Variants Are Associated With Hyperferritinemia and Increased Liver Iron in Patients With NAFLD,” Journal of Hepatology 75, no. 3 (2021): 506–513. 33774058 10.1016/j.jhep.2021.03.014

[21] A. T. Merryweather‐Clarke , E. Cadet , A. Bomford , et al., “Digenic Inheritance of Mutations in HAMP and HFE Results in Different Types of Haemochromatosis,” Human Molecular Genetics 12, no. 17 (2003): 2241–2247. 12915468 10.1093/hmg/ddg225

[22] S. Jacolot , G. Le Gac , V. Scotet , I. Quere , C. Mura , and C. Ferec , “HAMP as a Modifier Gene That Increases the Phenotypic Expression of the HFE pC282Y Homozygous Genotype,” Blood 103, no. 7 (2004): 2835–2840. 14670915 10.1182/blood-2003-10-3366

[23] G. Le Gac , V. Scotet , C. Ka , et al., “The Recently Identified Type 2A Juvenile Haemochromatosis Gene (HJV), a Second Candidate Modifier of the C282Y Homozygous Phenotype,” Human Molecular Genetics 13, no. 17 (2004): 1913–1918. 15254010 10.1093/hmg/ddh206

[24] V. L. Chen , Y. Chen , X. Du , S. K. Handelman , and E. K. Speliotes , “Genetic Variants That Associate With Cirrhosis Have Pleiotropic Effects on Human Traits,” Liver International 40, no. 2 (2020): 405–415. 31815349 10.1111/liv.14321 PMC7395656

[25] L. Valenti , “Uncovering the Genetics of Cirrhosis: New Plots for the Usual Suspects,” Liver International 40, no. 2 (2020): 281–282. 31967399 10.1111/liv.14333

[26] L. Valenti , P. Dongiovanni , A. Piperno , et al., “Alpha 1‐Antitrypsin Mutations in NAFLD: High Prevalence and Association With Altered Iron Metabolism but Not With Liver Damage,” Hepatology 44, no. 4 (2006): 857–864. 17006922 10.1002/hep.21329

[27] L. Braiterman , L. Nyasae , Y. Guo , R. Bustos , S. Lutsenko , and A. Hubbard , “Apical Targeting and Golgi Retention Signals Reside Within a 9‐Amino Acid Sequence in the Copper‐ATPase, ATP7B,” American Journal of Physiology. Gastrointestinal and Liver Physiology 296, no. 2 (2009): G433–G444. 19033537 10.1152/ajpgi.90489.2008 PMC2643914

[28] N. Maio , F. Polticelli , G. De Francesco , G. Rizzo , M. C. Bonaccorsi di Patti , and G. Musci , “Role of External Loops of Human Ceruloplasmin in Copper Loading by ATP7B and Ccc2p,” Journal of Biological Chemistry 285, no. 27 (2010): 20507–20513. 20430895 10.1074/jbc.M109.090027 PMC2898344

[29] N. E. Hellman and J. D. Gitlin , “Ceruloplasmin Metabolism and Function,” Annual Review of Nutrition 22 (2002): 439–458. 10.1146/annurev.nutr.22.012502.114457 12055353

[30] Z. K. Attieh , C. K. Mukhopadhyay , V. Seshadri , N. A. Tripoulas , and P. L. Fox , “Ceruloplasmin Ferroxidase Activity Stimulates Cellular Iron Uptake by a Trivalent Cation‐Specific Transport Mechanism,” Journal of Biological Chemistry 274, no. 2 (1999): 1116–1123. 9873059 10.1074/jbc.274.2.1116

[31] Y. Shiono , S. Wakusawa , H. Hayashi , et al., “Iron Accumulation in the Liver of Male Patients With Wilson's Disease,” American Journal of Gastroenterology 96, no. 11 (2001): 3147–3151. 11721763 10.1111/j.1572-0241.2001.05269.x

[32] K. Barada , A. El Haddad , M. Katerji , M. Jomaa , and J. Usta , “Wilson's Disease in Lebanon and Regional Countries: Homozygosity and Hepatic Phenotype Predominance,” World Journal of Gastroenterology 23, no. 36 (2017): 6715–6725. 29085216 10.3748/wjg.v23.i36.6715 PMC5643292

[33] K. Pak , S. Ordway , B. Sadowski , M. Canevari , and D. Torres , “Wilson's Disease and Iron Overload: Pathophysiology and Therapeutic Implications,” Clinical Liver Disease (Hoboken) 17, no. 2 (2021): 61–66. 10.1002/cld.986 PMC7916432 33680437

[34] T. Lefebvre , N. Reihani , R. Daher , et al., “Involvement of Hepcidin in Iron Metabolism Dysregulation in Gaucher Disease,” Haematologica 103, no. 4 (2018): 587–596. 29305416 10.3324/haematol.2017.177816 PMC5865418

[35] M. Regenboog , A. B. van Kuilenburg , J. Verheij , D. W. Swinkels , and C. E. Hollak , “Hyperferritinemia and Iron Metabolism in Gaucher Disease: Potential Pathophysiological Implications,” Blood Reviews 30, no. 6 (2016): 431–437. 27265538 10.1016/j.blre.2016.05.003

[36] D. A. Hughes and G. M. Pastores , “Gaucher Disease,” in GeneReviews((R)), ed. M. P. Adam , S. Bick , G. M. Mirzaa , R. A. Pagon , S. E. Wallace , and A. Amemiya (University of Washington, 1993).

[37] P. Stein , H. Yu , D. Jain , and P. K. Mistry , “Hyperferritinemia and Iron Overload in Type 1 Gaucher Disease,” American Journal of Hematology 85, no. 7 (2010): 472–476. 20575041 10.1002/ajh.21721 PMC2895498

[38] I. Motta , P. Delbini , N. Scaramellini , et al., “Enzyme Replacement Therapy Improves Erythropoiesis and Iron Dysregulation in Gaucher Disease,” Annals of Hematology 103, no. 12 (2024): 5113–5121. 39370488 10.1007/s00277-024-05918-2

