# Supplementary material for: Clinical Exome Sequencing in Unexplained Hyperferritinemia Reveals Digenic and Oligogenic Inheritance Beyond Iron Homeostasis
Source: Liver Int. 2026 Apr 12;46(5):e70646. doi: 10.1111/liv.70646 (PMC13071257; doi:10.1111/liv.70646)
Supplement: Supplementary file 1 — Table S1: List of genes included in variant filtering by panel and Human Phenotype Ontology annotation. Table S2: Clinical exome sequencing results restricted to likely pathogenic and pathogenic variants (N = 108). Table S3: Clinical exome sequencing results in patients without monogenic HFE disease (N = 57). Table S4: Genetic architecture according to variant classification (N = 108). Table S5: Gene‐level variant distribution across the study population (VUS, likely pathogenic and pathogenic; N = 108). Table S6: Gene co‐occurrence patterns suggestive of digenic or oligogenic inheritance (VUS, likely pathogenic and pathogenic). Table S7: Gene‐level variant distribution restricted to likely pathogenic and pathogenic variants (N = 108). Table S8: Gene co‐occurrence patterns restricted to likely pathogenic and pathogenic variants. Table S9: Variant‐level summary across the study population (VUS, likely pathogenic and pathogenic; N = 108) Table S10: Variant co‐occurrence patterns (VUS, likely pathogenic and pathogenic). Table S11: Variant‐level summary restricted to likely pathogenic and pathogenic variants (N = 108). Table S12: Variant co‐occurrence patterns restricted to likely pathogenic and pathogenic variants. Table S13: Patient classification by functional gene pathway. Figure S1: Gene co‐occurrence heatmap restricted to likely pathogenic and pathogenic variants. Figure S2: Functional pathway distribution including all retained variants. Figure S3: Pairwise pathway overlaps including all retained variants. Figure S4: Functional pathway distribution restricted to likely pathogenic and pathogenic variants. Figure S5: Pairwise pathway overlaps restricted to likely pathogenic and pathogenic variants. Figure S6: Iron metabolism parameters by functional pathway including all retained variants. Figure S7: Iron metabolism parameters in patients with digenic HFE/SERPINA1, HFE/ATP7B and SERPINA1/HBB combinations compared with monogenic HFE genotypes. [file LIV-46-0-s001.docx]

**SUPPLEMENTARY APPENDIX**

**Title**

Clinical Exome Sequencing in Unexplained Hyperferritinemia Reveals Digenic and Oligogenic Inheritance Beyond Iron Homeostasis

**Short title**

Exome Sequencing in Hyperferritinemia

**Authors**

Paul Morel*^1,2^; Maël Silva Rodriguez, MD, MSc*^1,2,3,4^; Cyriaque Benmouffek*^1,2^; Betul Goksen, MSc^1,2^; Céline Chéry, PhD^1,2^; Vincent Haghnejad, MD, MSc^5^; Mouni Bensenane, MD, MSc^5^; François Feillet, MD, PhD^2,3,4,6^; Jean-Louis Guéant, MD, DSc^7^; Farès Namour, MD, PhD^1,2,3,4^; Jean-Pierre Bronowicki, MD, PhD^2,3,4,5^; Abderrahim Oussalah, MD, PhD^1,2,3,4^

**These authors contributed equally to this work.*

1 Department of Genomic Medicine, Division of Biochemistry, Molecular Biology, and Nutrition, University Hospital of Nancy, F-54000 Nancy, France

2 Reference Center for Inborn Errors of Metabolism (ORPHA67872), University Hospital of Nancy, F-54000 Nancy, France

3 Faculty of Medicine of Nancy, University of Lorraine, F-54000 Nancy, France

4 INSERM UMR_S 1256, Nutrition, Genetics, and Environmental Risk Exposure (NGERE), F-54000 Nancy, France

5 Department of Gastroenterology and Liver Diseases, University Hospital of Nancy, Nancy, France

6 Department of Pediatrics, University Hospital of Nancy, Nancy, France

7 Joan Klein Jacobs Center for Precision Nutrition and Health, Cornell University, Ithaca, NY, USA

**Corresponding author**

Prof. Abderrahim Oussalah, MD, PhD, FEBGH

Department of Genomic Medicine, Division of Biochemistry, Molecular Biology, and Nutrition

University Hospital of Nancy

INSERM UMR_S 1256, Nutrition, Genetics, and Environmental Risk Exposure (NGERE)

Faculty of Medicine of Nancy

9 Avenue de la Forêt de Haye, Nancy, France

Telephone: +33 3 83 15 36 29

E-mail: abderrahim.oussalah@univ-lorraine.fr

**TABLE OF CONTENTS**

[SUPPLEMENTARY METHODS 5](#_Toc224198066)

[Data collection 5](#_Toc224198067)

[French framework for genetic diagnosis 5](#_Toc224198068)

[Ethics 5](#_Toc224198069)

[Data availability 5](#_Toc224198070)

[Next-generation sequencing: bioinformatics analysis 6](#_Toc224198071)

[Functional classification of genes 7](#_Toc224198072)

[Patient allocation to functional pathway groups 8](#_Toc224198073)

[Statistical analysis 9](#_Toc224198074)

[Artificial intelligence disclosure 9](#_Toc224198075)

[SUPPLEMENTARY TABLES 11](#_Toc224198076)

[Supplementary Table S1. List of genes included in variant filtering by panel and Human Phenotype Ontology annotation 11](#_Toc224198077)

[Supplementary Table S2. Clinical Exome Sequencing Results Restricted to Likely Pathogenic and Pathogenic Variants (N=108). 13](#_Toc224198078)

[Supplementary Table S3. Clinical Exome Sequencing Results in Patients Without Monogenic *HFE* Disease (N=57). 16](#_Toc224198079)

[Supplementary Table S4. Genetic Architecture According to Variant Classification (N=108) 19](#_Toc224198080)

[Supplementary Table S5. Gene-Level Variant Distribution Across the Study Population (VUS, Likely Pathogenic, and Pathogenic; N=108) 20](#_Toc224198081)

[Supplementary Table S6. Gene Co-occurrence Patterns Suggestive of Digenic or Oligogenic Inheritance (VUS, Likely Pathogenic, and Pathogenic) 21](#_Toc224198082)

[Supplementary Table S7. Gene-Level Variant Distribution Restricted to Likely Pathogenic and Pathogenic Variants (N=108) 22](#_Toc224198083)

[Supplementary Table S8. Gene Co-occurrence Patterns Restricted to Likely Pathogenic and Pathogenic Variants 23](#_Toc224198084)

[Supplementary Table S9. Variant-Level Summary Across the Study Population (VUS, Likely Pathogenic, and Pathogenic; N=108) 24](#_Toc224198085)

[Supplementary Table S10. Variant Co-occurrence Patterns (VUS, Likely Pathogenic, and Pathogenic) 26](#_Toc224198086)

[Supplementary Table S11. Variant-Level Summary Restricted to Likely Pathogenic and Pathogenic Variants (N=108) 28](#_Toc224198087)

[Supplementary Table S12. Variant Co-occurrence Patterns Restricted to Likely Pathogenic and Pathogenic Variants 30](#_Toc224198088)

[Supplementary Table S13. Patient Classification by Functional Gene Pathway 31](#_Toc224198089)

[SUPPLEMENTARY FIGURES 33](#_Toc224198090)

[Supplementary Figure S1. Gene Co-occurrence Heatmap Restricted to Likely Pathogenic and Pathogenic Variants 33](#_Toc224198091)

[Supplementary Figure S2. Functional Pathway Distribution Including All Retained Variants 34](#_Toc224198092)

[Supplementary Figure S3. Pairwise Pathway Overlaps Including All Retained Variants 35](#_Toc224198093)

[Supplementary Figure S4. Functional Pathway Distribution Restricted to Likely Pathogenic and Pathogenic Variants 36](#_Toc224198094)

[Supplementary Figure S5. Pairwise Pathway Overlaps Restricted to Likely Pathogenic and Pathogenic Variants 37](#_Toc224198095)

[Supplementary Figure S6. Iron Metabolism Parameters by Functional Pathway Including All Retained Variants 38](#_Toc224198096)

[Supplementary Figure S7. Iron metabolism parameters in patients with digenic *HFE*/*SERPINA1*, *HFE*/*ATP7B*, and *SERPINA1*/*HBB* combinations compared with monogenic *HFE* genotypes 39](#_Toc224198097)

#

# **SUPPLEMENTARY METHODS**

# **Data collection**

We extracted administrative and clinical data from electronic health records through chart review using DxCare software (Dedalus France, Le Plessis Robinson, France). These data included the referring institution and clinical setting, demographic characteristics (age at CES prescription, sex), clinical indication for testing, relevant medical history including alcohol consumption, body mass index, and baseline *HFE* genotyping status when available. Laboratory data encompassed iron metabolism parameters (serum ferritin, serum iron, transferrin, transferrin saturation), liver biochemistry (aspartate aminotransferase, alanine aminotransferase, gamma-glutamyltransferase, alkaline phosphatase, albumin, prothrombin time), metabolic parameters (fasting glucose, triglycerides, total cholesterol), complete blood count, and renal function tests. When available, hepatic imaging data were also collected, including iron concentration measured by magnetic resonance imaging and liver stiffness assessed by transient elastography. Genomic data were obtained from molecular diagnosis reports and included the number of retained variants, variant annotation according to Human Genome Variation Society nomenclature (coding DNA and protein sequences), the affected gene, ACMG classification, and zygosity status.

# **French framework for genetic diagnosis**

In France, patients with suspected genetic disorders are referred to specialized reference centers established under the French National Plan for Rare Diseases. The Reference Center for Inborn Errors of Metabolism at the University Hospital of Nancy (ORPHA67872) serves as one such center, receiving patients referred by their treating physicians for genetic evaluation. Written informed consent is obtained prior to genetic testing, which is fully reimbursed by the French health care system ^1-3^.

# **Ethics**

The Ethics Committee of the University Hospital of Nancy approved the study protocol (ID: 2020/264). The Nancy Biochemical Database is registered with the French National Commission on Informatics and Liberty (CNIL N°1763197v0).^1-3^ Written informed consent was obtained from all patients prior to inclusion.

# **Data availability**

Under French law, genetic data are subject to specific regulatory restrictions that preclude their sharing outside the institution where they were generated. Anonymized clinical and biochemical data from this study may be available to qualified researchers upon reasonable request. Requests will be reviewed by the Institutional Review Board of the University Hospital of Nancy and require submission of a research proposal, a statistical analysis plan, and execution of a data-sharing agreement.

# **Next-generation sequencing: bioinformatics analysis**

Bioinformatics analysis was performed as previously described ^4-7^. The Burrows-Wheeler Aligner (BWA) software package was used to map all reads to GRCh37/hg19 annotation using the BWA-MEM algorithm. PCR duplicates were removed from alignments using the Picard MarkDuplicates tool (http://broadinstitute.github.io/picard). Indels were realigned using the Genome Analysis Toolkit (GATK) ^8^. Single nucleotide variants and short indels were called using the GATK HaplotypeCaller software ^8^.

Two successive bioinformatics pipelines were used for variant analysis over the study period. Before 2022, an in-house pipeline was employed with the following strategy for filtering (gene panels detailed below) and prioritizing genetic variants: Step 1, import all retrieved genetic variants; Step 2, retain only low-frequency and rare variants by excluding variants with a reported alternative allele frequency greater than 5% in the Exome Aggregation Consortium and the Genome Aggregation Database (gnomAD Exomes). The aim of step 2 was to retain rare (alternative allele frequency less than 1%) and low-frequency (alternative allele frequency less than 5%) variants that exhibit relatively large effects on disease risk, such as those involved in severe monogenic diseases ^9^; Step 3, exclude intronic variants other than splice donor, splice acceptor, or splice region variants, and exclude synonymous variants; Step 4, prioritize genetic variants for their association with the studied phenotype using the SVS-PhoRank gene ranking algorithm based on the Phevor algorithm ^9^. The Phevor algorithm scores ontology terms based on their proximity to the phenotypes of the patient. The SVS-PhoRank score was calculated according to RefSeq Genes 105v2, NCBI gene source, using the following ontologies: Human Phenotype Ontology, Gene Ontology, and OMIM Phenotype Ontology. Functional variant annotation was performed using the following tools: MutationTaster ^10^, Functional Analysis through Hidden Markov Models (FATHMM) ^11^, MetaSVM/MetaLR scores ^12^, Sorting Tolerant From Intolerant (SIFT) ^13^, Protein Variation Effect Analyzer (Provean) ^14^, and deleterious annotation of genetic variants using neural networks (DANN) ^15^. Variant pathogenicity was reported according to American College of Medical Genetics and Genomics (ACMG) criteria ^16^. All retained and top-prioritized variants were systematically assessed for their ClinVar annotation ^17^. All genetic variants retained in the molecular diagnosis report were required to meet high-quality metric criteria (read depth greater than 20, alt-read ratio greater than 0.2, genotype quality score of 99). Bioinformatics analyses for the in-house pipeline were performed using the SNP and Variation Suite (v8.9.0; Golden Helix, Inc., Bozeman, MT, USA).

Beginning in 2022, we implemented the Franklin Premium platform (Franklin by Genoox; https://franklin.genoox.com) as a clinical decision support tool for variant prioritization and annotation. Franklin adheres to ACMG and Association for Molecular Pathology standards and guidelines for variant classification. The platform incorporates a proprietary artificial intelligence (AI)-based engine for both variant classification and phenotype-driven prioritization (https://u2l.fr/l7qmzl). The AI-based classification algorithms evaluate classification criteria by incorporating gene-specific evidence from more than 100 different sources, including variant databases (ClinVar, gnomAD), gene curation resources (ClinGen, Uniprot), functional annotation tools, population frequency databases, and in silico prediction algorithms. The platform also integrates genotype-to-phenotype associations from an extensive range of databases, including Orphanet, Monarch, Human Phenotype Ontology (HPO), and DECIPHER, and scores the relationships between patient clinical terms and genes using HPO terms. The relationships between patient clinical terms and genes are scored and ranked, with the prioritization algorithm factoring in the strength of connections between patient phenotypes and genes. The prioritization algorithm also considers possible inheritance models per variant and integrates this information with supported gene inheritance models derived from published and curated sources. Franklin also incorporates novel evidence through a text-mining engine that scans public literature. Large structural variations were also assessed using this platform.

Variant filtering was performed using established gene panels, including the French national network for rare liver diseases panel FILFOIE (Filfoie_Panel Hepato_V8, 183 genes; https://www.filfoie.com/) and the Genomics England PanelApp panel for iron metabolism disorders (versions 2.3 to 3.3, 27 genes; https://panelapp.genomicsengland.co.uk/). The union of these two panels, excluding shared genes, comprises 199 genes. For the present study, we used the following four HPO annotations: increased circulating ferritin concentration (HP:0003281), elevated transferrin saturation (HP:0012463), elevated hepatic iron concentration (HP:0012465), and abnormality of iron homeostasis (HP:0011031). These four HPO annotations collectively correspond to 73 genes after exclusion of shared genes. Combining gene panels and HPO-related genes, the total number of genes of interest is 251 (Supplementary Table S1).

In both pipelines (SVS-based and Franklin-based), final variant classification according to ACMG/AMP criteria remained under the sole responsibility of the clinical molecular geneticists involved in the diagnosis. Variant classification was based on the molecular diagnosis reports issued at the time of testing; no systematic re-evaluation was performed for the purpose of this study. All genetic variants retained in the bioinformatics analysis were confirmed by visual inspection of the read-level data from BAM files. Sanger confirmatory sequencing was performed in rare cases of variants with suboptimal quality metric indicators (e.g., splice donor or acceptor variants, homozygous variants).

# **Functional classification of genes**

All variants reported in the molecular diagnosis reports as part of routine clinical care were extracted for this study. Genes harboring at least one variant classified as VUS, LP, or P were then classified a posteriori into four functional groups according to their roles in iron homeostasis and the pathophysiological mechanisms through which they contribute to hyperferritinemia. The first group, designated IRON-SENS (systemic iron sensing and hepcidin regulation), comprises genes whose protein products participate in the hepatic iron-sensing complex and regulate hepcidin transcription. This group includes *HFE* (homeostatic iron regulator), *HJV* (hemojuvelin BMP co-receptor), *HAMP* (hepcidin antimicrobial peptide), and *TFR2* (transferrin receptor 2). Pathogenic variants in these genes lead to hereditary hemochromatosis through impaired hepcidin regulation. The second group, IRON-TRANS (iron transport and storage), encompasses genes involved in extracellular iron trafficking and intracellular iron storage. This group includes *TF* (transferrin), *FTL* (ferritin light chain), *CP* (ceruloplasmin), and *ATP7B* (ATPase copper transporting beta). Variants in *FTL* are responsible for hereditary hyperferritinemia-cataract syndrome, whereas *ATP7B* deficiency, which causes Wilson disease, disrupts iron metabolism secondarily through impaired ceruloplasmin synthesis. The third group, HEPATO-METAB (hepatic metabolism and lysosomal storage), includes genes associated with hepatocellular dysfunction and lysosomal storage disorders that can manifest with secondary hyperferritinemia. This group comprises *SERPINA1* (serpin family A member 1), *GBA* (glucosylceramidase beta 1), *CFTR* (cystic fibrosis transmembrane conductance regulator), *HNF1B* (HNF1 homeobox B), and *BCS1L* (BCS1 homolog, ubiquinol-cytochrome c reductase complex chaperone). Elevated ferritin in these disorders is typically accompanied by normal or low transferrin saturation. The fourth group, ERYTHRO-HEME (erythropoiesis and heme biosynthesis), includes genes implicated in erythrocyte production and heme synthesis pathways. This group comprises *HBB* (hemoglobin subunit beta), *ANK1* (ankyrin 1), *SLC4A1* (solute carrier family 4 member 1), and *UROD* (uroporphyrinogen decarboxylase). These conditions share a common feature of dysregulated erythropoiesis or heme metabolism, which contributes to iron maldistribution.

# **Patient allocation to functional pathway groups**

For pathway-based analyses, each patient was assigned to a single primary functional pathway group to avoid double counting in between-group comparisons. The allocation algorithm followed a hierarchical decision process based on variant characteristics. First, all variants classified as VUS, LP, or P were extracted for each patient. Variants classified as benign or likely benign were excluded from pathway assignment. Each qualifying variant was mapped to its corresponding functional pathway group based on the gene involved. For patients harboring variants in a single pathway, assignment was straightforward: the patient was allocated to that pathway. For patients with variants spanning multiple pathways, the following prioritization rules were applied. If a patient carried a homozygous variant together with heterozygous variants in different genes affecting multiple pathways, the pathway containing the homozygous variant was designated as the primary pathway, reflecting the expected greater phenotypic contribution of biallelic variants. In the absence of homozygous variants, patients with multi-pathway involvement were assigned to the pathway containing the variant with the highest ACMG classification (P prioritized over LP, LP prioritized over VUS). When variants across pathways shared the same ACMG classification, the first-listed variant in the molecular diagnosis report determined the primary pathway assignment. Zygosity status was classified into five categories for visualization purposes: heterozygous (HTZ, single heterozygous variant), homozygous (HMZ, biallelic variants in a single gene), homozygous with additional heterozygous variant (HMZ + HTZ, homozygous variant plus heterozygous variant in a different gene), double heterozygous in the same gene (two heterozygous variants in *cis* or *trans* configuration within the same gene), and multi-locus heterozygous (heterozygous variants in two or more different genes). Patients without any qualifying variant (VUS, LP, or P) in the genes included in the four functional groups were classified as negative for the pathway-based analyses. This classification scheme enabled mutually exclusive group assignment while preserving information about the underlying genetic architecture through the zygosity categorization.

# **Statistical analysis**

This study was exploratory in nature; no a priori sample size calculation based on statistical power was performed, and P values are reported for descriptive purposes without adjustment for multiple comparisons. Categorical variables are presented as counts and percentages. Given the non-normal distribution of the data, continuous variables are expressed as medians with interquartile ranges; 95% confidence intervals for medians were estimated using bootstrap resampling (10,000 iterations). Comparisons of continuous variables across functional pathway groups were performed using the Kruskal-Wallis test, with effect sizes estimated using eta-squared (η^2^ < 0.01 indicates a negligible effect, 0.01 ≤ η^2^ < 0.06 a small effect, 0.06 ≤ η^2^ < 0.14 a medium effect, and η^2^ ≥ 0.14 a large effect). Given the descriptive and exploratory nature of this study, post-hoc pairwise comparisons were not performed; the primary objective was to characterize the distribution of phenotypic variables across pathway groups rather than to identify specific between-group differences. Gene and variant co-occurrence patterns were visualized using UpSet plots and heatmaps. The distribution of patients across functional pathways and their overlaps was visualized using bar charts and pairwise Venn diagrams. For the purpose of these analyses, patients were classified according to their genetic architecture as monogenic, digenic, or oligogenic. To visualize the combined effects of zygosity and multigenic architecture on biological phenotypes, including iron metabolism parameters and hepatic iron concentration, we used a data visualization approach based on violin plots with individual data points stratified by zygosity category: heterozygous (HTZ), homozygous (HMZ), homozygous with additional heterozygous variant (HMZ + HTZ), double heterozygous in the same gene (*cis* or *trans*), and multi-locus heterozygous. Pathway comparison analyses were conducted in two ways: first, including all patients with retained variants regardless of ACMG classification (VUS, LP, or P), and second, restricting the analysis to patients with LP or P variants only. All statistical analyses were performed using Python version 3.12 with the pandas, numpy, scipy, matplotlib, matplotlib-venn, seaborn, and upsetplot libraries. Python scripts were developed and executed using PyCharm Professional Edition (version 2025.1; JetBrains, Prague, Czech Republic).

# **Artificial intelligence disclosure**

The manuscript was written by the authors. No artificial intelligence tool was used to generate text or images de novo. Claude (Anthropic) was used during manuscript preparation for quality control, methodological clarification based exclusively on content provided by the authors. No autonomous data analysis was performed. The authors take full responsibility for the content of the manuscript.

# **SUPPLEMENTARY TABLES**

# **Supplementary Table S1. List of genes included in variant filtering by panel and Human Phenotype Ontology annotation**

| **Source** | **Genes** |
| --- | --- |
| Genomics England PanelApp panel for iron metabolism disorders (n = 27) | *ABCB7*, *ACVR1*, *ALAS2*, *ATP7B*, *BMP6*, *CDAN1*, *CP*, *CYBRD1*, *FECH*, *FTH1*, *FTL*, *GBA*, *GLRX5*, *HAMP*, *HEPH*, *HFE*, *HJV*, *SEC23B*, *SERPINA1*, *SLC11A2*, *SLC25A38*, *SLC40A1*, *STAB1*, *STEAP3*, *TF*, *TFR2*, *TMPRSS6* |
| French national network for rare liver diseases panel FILFOIE (n = 183) | *ABCB11*, *ABCB4*, *ABCC12*, *ABCC2*, *ABCG5*, *ABCG8*, *ACOX2*, *ADK*, *AGL*, *AIRE*, *AKR1D1*, *ALDOB*, *ALG8*, *AMACR*, *AP1S1*, *ATP7A*, *ATP7B*, *ATP8B1*, *AVEN*, *BAAT*, *BCS1L*, *BMP2*, *C4orf54*, *CC2D2A*, *CCBE1*, *CFTR*, *CLDN1*, *CLDN6*, *CLDN9*, *COG7*, *CP*, *CPT1A*, *CTC1*, *CYP27A1*, *CYP7A1*, *CYP7B1*, *DCDC2*, *DGUOK*, *DHCR7*, *DKC1*, *DLD*, *F11R*, *FAH*, *FBP1*, *FOCAD*, *FTH1*, *FUT2*, *G6PC*, *GALE*, *GALM*, *GALT*, *GANAB*, *GBA*, *GBE1*, *GFM1*, *GIMAP5*, *GPBAR1*, *GYG2*, *GYS2*, *HADHA*, *HAMP*, *HFE*, *HJV*, *HNF1A*, *HNF1B*, *HSD17B4*, *HSD3B7*, *IARS*, *IFT140*, *IFT172*, *INVS*, *ITCH*, *JAG1*, *KCNN3*, *KIF12*, *KIF3B*, *LAMTOR1*, *LAMTOR3*, *LARS*, *LFNG*, *LIPA*, *LRP5*, *LSR*, *MARS*, *MFNG*, *MKS1*, *MPI*, *MPV17*, *MTOR*, *MYO5B*, *NBAS*, *NEK8*, *NFAM1*, *NOTCH1*, *NOTCH2*, *NPC1*, *NPC2*, *NPHP1*, *NPHP3*, *NPHP4*, *NR1H4*, *PGM1*, *PHKA2*, *PHKB*, *PHKG2*, *PKD1*, *PKD1L1*, *PKD2*, *PKHD1*, *PLEC*, *PNPLA3*, *POGLUT1*, *POLG*, *PPM1F*, *PPP1R15B*, *PRKAG1*, *PRKCSH*, *PTEN*, *PYGL*, *RAB10*, *RAB11A*, *RDX*, *RFNG*, *RINT1*, *RRAGC*, *RTEL1*, *SCO1*, *SCP2*, *SCYL1*, *SEC61B*, *SEC63*, *SEMA4D*, *SEMA7A*, *SERAC1*, *SERPINA1*, *SI*, *SLC10A1*, *SLC10A2*, *SLC11A2*, *SLC25A13*, *SLC27A5*, *SLC2A2*, *SLC2A5*, *SLC30A10*, *SLC37A4*, *SLC40A1*, *SLC4A2*, *SLC51A*, *SLC51B*, *SLC5A1*, *SLCO1B1*, *SLCO1B3*, *SMPD1*, *SOX9*, *STN1*, *TALDO1*, *TERT*, *TFR2*, *THBS2*, *TJP2*, *TKFC*, *TMEM216*, *TMEM30A*, *TMEM67*, *TRMU*, *TSC2*, *TTC17*, *TTC26*, *TTC37*, *TUFM*, *UGT1A1*, *UNC45A*, *USP53*, *UTP4*, *VIL1*, *VIPAS39*, *VPS33B*, *VPS50*, *WDR35*, *WDR60*, *WDR83OS*, *YARS*, *ZFYVE19* |
| HP:0003281, increased circulating ferritin concentration (n = 58) | *ALK*, *BCS1L*, *BMP2*, *BMP6*, *CP*, *CPOX*, *FOCAD*, *FTH1*, *FTL*, *GBA1*, *GLRX5*, *HACE1*, *HAMP*, *HAVCR2*, *HBB*, *HFE*, *HJV*, *HLA-DRB1*, *HMOX1*, *IFIH1*, *IFNG*, *IFT56*, *IKZF2*, *IL6*, *ITK*, *KARS1*, *KCNN4*, *LACC1*, *LIN28B*, *LMO1*, *LYST*, *MCM10*, *MIF*, *MPV17*, *MYCN*, *NLRC4*, *PHOX2B*, *PIEZO1*, *PIGA*, *PIK3CG*, *PKLR*, *PRF1*, *PSMB9*, *PUS1*, *RC3H1*, *SCARB2*, *SLC19A1*, *SLC25A38*, *SLC40A1*, *SLC4A1*, *SLC7A7*, *STAT2*, *STEAP3*, *STX11*, *STXBP2*, *TFR2*, *UNC13D*, *XIAP* |
| HP:0012463, elevated transferrin saturation (n = 11) | *BMP6*, *FTH1*, *HAMP*, *HFE*, *HJV*, *PIGA*, *PKLR*, *SLC25A38*, *SLC40A1*, *STEAP3*, *TFR2* |
| HP:0012465, elevated hepatic iron concentration (n = 12) | *BCS1L*, *BMP6*, *CP*, *FARS2*, *FOCAD*, *FTH1*, *FTL*, *GLRX5*, *HBB*, *PIGA*, *SLC11A2*, *STEAP3* |
| HP:0011031, abnormality of iron homeostasis (n = 30) | *ABCD3*, *ALAS2*, *BCS1L*, *BMP2*, *BMP6*, *CARD9*, *COL7A1*, *CP*, *FOXP1*, *FTH1*, *FTL*, *HAMP*, *HBB*, *HFE*, *HJV*, *KIF23*, *PIGA*, *PKLR*, *RACGAP1*, *SKIC2*, *SKIC3*, *SLC11A2*, *SLC25A38*, *SLC30A10*, *SLC40A1*, *STAB1*, *STEAP3*, *TFR2*, *TMPRSS6*, *TRNT1* |

Abbreviation: HPO, Human Phenotype Ontology.

HPO annotations combined (excluding shared genes): 73 genes. Panels combined (excluding shared genes): 199 genes. Total genes of interest (all sources combined, excluding duplicates): 251 genes.

The clinical exome sequencing panel covers approximately 6699 genes (TruSight One Sequencing Panels Manifest File Download, https://support.illumina.com/downloads/trusight_one_sequencing_panel_product_file.html).

# **Supplementary Table S2. Clinical Exome Sequencing Results Restricted to Likely Pathogenic and Pathogenic Variants (N=108)**

| **Category** |  | **No. (%)** |
| --- | --- | --- |
| **Baseline *HFE*** **Genetic Testing** |  |  |
| Not performed |  | 67 (62.0) |
| Negative |  | 19 (17.6) |
| H63D heterozygous |  | 11 (10.2) |
| C282Y heterozygous |  | 6 (5.6) |
| C282Y/H63D compound heterozygous |  | 4 (3.7) |
| H63D homozygous |  | 1 (0.9) |
| **Distribution of patients by LP/P variant count** |  |  |
| 0 |  | 64 (59.3) |
| 1 |  | 34 (31.5) |
| 2 |  | 9 (8.3) |
| 3 |  | 1 (0.9) |
| **Primary Variant — LP/P Only (Variant 1)** |  |  |
| Variant 1 — Gene |  |  |
| None identified |  | 64 (59.3) |
| *HFE* |  | 21 (19.4) |
| *SERPINA1* |  | 6 (5.6) |
| *ATP7B* |  | 5 (4.6) |
| *CP* |  | 2 (1.9) |
| *GBA* |  | 2 (1.9) |
| *ANK1* |  | 1 (0.9) |
| *BCS1L* |  | 1 (0.9) |
| *FTL* |  | 1 (0.9) |
| *HBB* |  | 1 (0.9) |
| *HNF1B* |  | 1 (0.9) |
| *SLC4A1* |  | 1 (0.9) |
| *TF* |  | 1 (0.9) |
| *UROD* |  | 1 (0.9) |
| Variant 1 — Zygosity |  |  |
| Heterozygous |  | 41 (38.0) |
| Homozygous |  | 3 (2.8) |
| Variant 1 — ACMG Classification |  |  |
| Pathogenic |  | 36 (33.3) |
| Likely pathogenic |  | 8 (7.4) |
| Variant 1 — HGVS Nomenclature | ACMG |  |
| *HFE*: NM_000410.4:c.845G>A (p.Cys282Tyr) | P | 21 (19.4) |
| *SERPINA1*: NM_000295.5:c.1096G>A (p.Glu366Lys) | P | 3 (2.8) |
| *SERPINA1*: NM_000295.5:c.863A>T (p.Glu288Val) | P | 3 (2.8) |
| *GBA*: NM_000157.4:c.1448T>C (p.Leu483Pro) | P | 2 (1.9) |
| *ANK1*: NM_000037.4:c.5504_5507del (p.Ile1835Thrfs*16) | P | 1 (0.9) |
| *ATP7B*: NM_000053.4:c.1512dup (p.Asn505*) | P | 1 (0.9) |
| *ATP7B*: NM_000053.4:c.230dup (p.Asp78Glyfs*85) | P | 1 (0.9) |
| *ATP7B*: NM_000053.4:c.2605G>A (p.Gly869Arg) | LP | 1 (0.9) |
| *ATP7B*: NM_000053.4:c.2978C>T (p.Thr993Met) | LP | 1 (0.9) |
| *ATP7B*: NM_000053.4:c.3275C>T (p.Thr1092Met) | LP | 1 (0.9) |
| *BCS1L*: NM_004328.5:c.460+2T>C (Splice Donor) | P | 1 (0.9) |
| *CP*: NM_000096.4:c.2476_2495del (p.Asn826Leufs*21) | LP | 1 (0.9) |
| *CP*: NM_000096.4:c.2520_2523del (p.Thr841Argfs*52) | LP | 1 (0.9) |
| *FTL*: NM_000146.4:c.89C>T (p.Thr30Ile) | LP | 1 (0.9) |
| *HBB*: NM_000518.5:c.118C>T (p.Gln40*) | P | 1 (0.9) |
| *HNF1B* (del 17q12): NC_000017.10:g.(34892941_36104886)del† | P | 1 (0.9) |
| *SLC4A1*: NM_000342.4:c.448C>T (p.Arg150*) | P | 1 (0.9) |
| *TF*: NM_001063.4:c.1203+1G>T (Splice donor) | LP | 1 (0.9) |
| *UROD*: NM_000374.5:c.390G>T (p.Glu130Asp) | LP | 1 (0.9) |
| **Secondary Variant — LP/P Only (Variant 2)** |  |  |
| Variant 2 — Gene |  |  |
| None identified |  | 98 (90.7) |
| *SERPINA1* |  | 8 (7.4) |
| *ATP7B* |  | 1 (0.9) |
| *GBA* |  | 1 (0.9) |
| Variant 2 — Zygosity |  |  |
| Heterozygous |  | 10 (9.3) |
| Variant 2 — ACMG Classification |  |  |
| Pathogenic |  | 9 (8.3) |
| Likely pathogenic |  | 1 (0.9) |
| Variant 2 — HGVS Nomenclature | ACMG |  |
| *SERPINA1*: NM_000295.5:c.1096G>A (p.Glu366Lys) | P | 4 (3.7) |
| *SERPINA1*: NM_000295.5:c.863A>T (p.Glu288Val) | P | 4 (3.7) |
| *ATP7B*: NM_000053.4:c.3053C>T (p.Ala1018Val) | P | 1 (0.9) |
| *GBA*: NM_000157.4:c.247C>T (p.Arg83Cys) | LP | 1 (0.9) |
| **Tertiary Variant — LP/P Only (Variant 3)** |  |  |
| Variant 3 — Gene |  |  |
| None identified |  | 107 (99.1) |
| *HBB* |  | 1 (0.9) |
| Variant 3 — Zygosity |  |  |
| Heterozygous |  | 1 (0.9) |
| Variant 3 — ACMG Classification |  |  |
| Pathogenic |  | 1 (0.9) |
| Variant 3 — HGVS Nomenclature | ACMG |  |
| *HBB*: NM_000518.5:c.126_129del (p.Phe42Leufs*19) | P | 1 (0.9) |
| **Quaternary Variant — LP/P Only (Variant 4)** |  |  |
| Variant 4 — Gene |  |  |
| None identified |  | 108 (100) |

Abbreviations: ACMG, American College of Medical Genetics and Genomics; *HFE*, homeostatic iron regulator gene; HGVS, Human Genome Variation Society; LP, likely pathogenic; P, pathogenic.

Data are presented as No. (%) unless otherwise indicated. Variant details are restricted to ACMG likely pathogenic and pathogenic variants only.

Percentages are calculated based on the number of patients with available data for each variable. Variant details (Gene, Zygosity, ACMG Classification, HGVS Nomenclature) are filtered to include only ACMG likely pathogenic and pathogenic variants. Patients without identified variants ("None identified") are included in the denominator for percentage calculations. HGVS nomenclature follows Human Genome Variation Society guidelines. Gene symbols are presented in italics according to HUGO Gene Nomenclature Committee guidelines.

†The *HNF1B* variant is a 1.21 Mb deletion at 17q12 (seq[GRCh37] del(17)(q12); chr17:34892941-36104886; NC_000017.10:g.(34892941_36104886)del) encompassing 17 loci: *HNF1B*, *PIGW*, *MRM1*, *DHRS11*, *LHX1*, *C17orf78*, *DDX52*, *ACACA*, *SNORA90*, *GGNBP2*, *TADA2A*, *SYNRG*, *AATF*, *MIR378J*, *DUSP14*, *LHX1-DT*, and *MIR2909*. The deletion was confirmed using chromosomal microarray analysis in an independent ISO 15189-accredited laboratory. The phenotypic effect was attributed to *HNF1B* haploinsufficiency based on ClinGen Haploinsufficiency score of 3, dominant inheritance pattern associated with hepatic manifestations (OMIM #614527, GeneReviews NBK401562, and reference ^18^), and high probability of loss-of-function intolerance (pLI = 1; o/e LOF upper = 0.17; DECIPHER HI score = 0.76). Other genes in the region have no established disease association, follow autosomal recessive inheritance (*PIGW*, *ACACA*), or lack evidence of haploinsufficiency.

# **Supplementary Table S3. Clinical Exome Sequencing Results in Patients Without Monogenic *HFE* Disease (N=57)**

| **Category** |  | **No. (%)** |
| --- | --- | --- |
| **Baseline *HFE*** **Genetic Testing** |  |  |
| Not performed |  | 39 (68.4) |
| Negative |  | 18 (31.6) |
| **Number of Variants Identified in the CES** |  |  |
| 0 |  | 36 (63.2) |
| 1 |  | 14 (24.6) |
| 2 |  | 7 (12.3) |
| **Primary Variant (Variant 1)** |  |  |
| Variant 1 — Gene |  |  |
| None identified |  | 36 (63.2) |
| *CP* |  | 5 (8.8) |
| *ATP7B* |  | 3 (5.3) |
| *SERPINA1* |  | 3 (5.3) |
| *GBA* |  | 2 (3.5) |
| *ANK1* |  | 1 (1.8) |
| *CFTR* |  | 1 (1.8) |
| *HBB* |  | 1 (1.8) |
| *HNF1B* |  | 1 (1.8) |
| *SLC4A1* |  | 1 (1.8) |
| *TF* |  | 1 (1.8) |
| *TFR2* |  | 1 (1.8) |
| *UROD* |  | 1 (1.8) |
| Variant 1 — Zygosity |  |  |
| Heterozygous |  | 21 (100.0) |
| Variant 1 — ACMG Classification |  |  |
| Pathogenic |  | 9 (42.9) |
| Likely pathogenic |  | 6 (28.6) |
| VUS |  | 6 (28.6) |
| Variant 1 — HGVS Nomenclature | ACMG |  |
| *SERPINA1*: NM_000295.5:c.1096G>A (p.Glu366Lys) | P | 3 (14.3) |
| *CP*: NM_000096.4:c.2684G>C (p.Gly895Ala) | VUS | 2 (9.5) |
| *ANK1*: NM_000037.4:c.5504_5507del (p.Ile1835Thrfs*16) | P | 1 (4.8) |
| *ATP7B*: NM_000053.4:c.230dup (p.Asp78Glyfs*85) | P | 1 (4.8) |
| *ATP7B*: NM_000053.4:c.2978C>T (p.Thr993Met) | LP | 1 (4.8) |
| *ATP7B*: NM_000053.4:c.3275C>T (p.Thr1092Met) | LP | 1 (4.8) |
| *CFTR*: NM_000492.4:c.2991G>C (p.Leu997Phe) | VUS | 1 (4.8) |
| *CP*: NM_000096.4:c.2476_2495del (p.Asn826Leufs*21) | LP | 1 (4.8) |
| *CP*: NM_000096.4:c.2520_2523del (p.Thr841Argfs*52) | LP | 1 (4.8) |
| *CP*: NM_000096.4:c.2799C>A (p.Asp933Glu) | VUS | 1 (4.8) |
| *GBA*: NM_000157.4:c.115+4A>C (Splice region, near donor) | VUS | 1 (4.8) |
| *GBA*: NM_000157.4:c.1448T>C (p.Leu483Pro) | P | 1 (4.8) |
| *HBB*: NM_000518.5:c.118C>T (p.Gln40*) | P | 1 (4.8) |
| *HNF1B* (del 17q12): NC_000017.10:g.(34892941_36104886)del† | P | 1 (4.8) |
| *SLC4A1*: NM_000342.4:c.448C>T (p.Arg150*) | P | 1 (4.8) |
| *TF*: NM_001063.4:c.1203+1G>T (Splice donor) | LP | 1 (4.8) |
| *TFR2*: NM_003227.4:c.1118G>A (p.Gly373Asp) | VUS | 1 (4.8) |
| *UROD*: NM_000374.5:c.390G>T (p.Glu130Asp) | LP | 1 (4.8) |
| **Secondary Variant (Variant 2)** |  |  |
| Variant 2 — Gene |  |  |
| None identified |  | 50 (87.7) |
| *SERPINA1* |  | 3 (5.3) |
| *GBA* |  | 1 (1.8) |
| *HAMP* |  | 1 (1.8) |
| *TF* |  | 1 (1.8) |
| *TFR2* |  | 1 (1.8) |
| Variant 2 — Zygosity |  |  |
| Heterozygous |  | 7 (100.0) |
| Variant 2 — ACMG Classification |  |  |
| Pathogenic |  | 3 (42.9) |
| VUS |  | 4 (57.1) |
| Variant 2 — HGVS Nomenclature | ACMG |  |
| *SERPINA1*: NM_000295.5:c.863A>T (p.Glu288Val) | P | 2 (28.6) |
| *GBA*: NM_000157.4:c.115+3G>A (Splice region, near donor) | VUS | 1 (14.3) |
| *HAMP*: NM_021175.4:c.212G>A (p.Gly71Asp) | VUS | 1 (14.3) |
| *SERPINA1*: NM_000295.5:c.1096G>A (p.Glu366Lys) | P | 1 (14.3) |
| *TF*: NM_001063.4:c.1925G>A (p.Arg642Gln) | VUS | 1 (14.3) |
| *TFR2*: NM_003227.4:c.1513G>A (p.Val505Met) | VUS | 1 (14.3) |
| **Tertiary Variant (Variant 3)** |  |  |
| Variant 3 — Gene |  |  |
| None identified |  | 57 (100) |
| **Quaternary Variant (Variant 4)** |  |  |
| Variant 4 — Gene |  |  |
| None identified |  | 57 (100) |

Abbreviations: ACMG, American College of Medical Genetics and Genomics; HFE, homeostatic iron regulator gene; HGVS, Human Genome Variation Society; LP, likely pathogenic; NGS, next-generation sequencing; P, pathogenic; VUS, variant of uncertain significance.

Data are presented as No. (%) unless otherwise indicated. N = 57 patients.

This table includes only patients without HFE gene variants in the NGS panel results. Percentages are calculated based on the number of patients with available data for each variable. HGVS nomenclature follows Human Genome Variation Society guidelines. Gene symbols are presented in italics according to HUGO Gene Nomenclature Committee guidelines.

†The *HNF1B* variant is a 1.21 Mb deletion at 17q12 (seq[GRCh37] del(17)(q12); chr17:34892941-36104886; NC_000017.10:g.(34892941_36104886)del) encompassing 17 loci: *HNF1B*, *PIGW*, *MRM1*, *DHRS11*, *LHX1*, *C17orf78*, *DDX52*, *ACACA*, *SNORA90*, *GGNBP2*, *TADA2A*, *SYNRG*, *AATF*, *MIR378J*, *DUSP14*, *LHX1-DT*, and *MIR2909*. The deletion was confirmed using chromosomal microarray analysis in an independent ISO 15189-accredited laboratory. The phenotypic effect was attributed to *HNF1B* haploinsufficiency based on ClinGen Haploinsufficiency score of 3, dominant inheritance pattern associated with hepatic manifestations (OMIM #614527, GeneReviews NBK401562, and reference ^18^), and high probability of loss-of-function intolerance (pLI = 1; o/e LOF upper = 0.17; DECIPHER HI score = 0.76). Other genes in the region have no established disease association, follow autosomal recessive inheritance (*PIGW*, *ACACA*), or lack evidence of haploinsufficiency.

# **Supplementary Table S4. Genetic Architecture According to Variant Classification (N=108)**

| **Genetic Architecture** | **All Retained Variants**  **(VUS, LP, P)** | **LP/P Variants Only** |
| --- | --- | --- |
| Negative | 36 (33.3) | 64 (59.3) |
| Monogenic | 50 (46.3) | 35 (32.4) |
| Digenic | 21 (19.4) | 8 (7.4) |
| Oligogenic | 1 (0.9) | 1 (0.9) |

Abbreviations: LP, likely pathogenic; P, pathogenic; VUS, variant of uncertain significance.

Monogenic indicates variants in a single gene. Digenic indicates variants in two genes. Oligogenic indicates variants in three or more genes.

# **Supplementary Table S5. Gene-Level Variant Distribution Across the Study Population (VUS, Likely Pathogenic, and Pathogenic; N=108)**

| **Gene** | **Functional Group** | **Total Patients** | **Homozygous** | **Heterozygous** | **% Homozygous** |
| --- | --- | --- | --- | --- | --- |
| *HFE* | IRON-SENS | 51 | 7 | 44 | 13.7 |
| *HJV* | IRON-SENS | 1 | 0 | 1 | 0.0 |
| *HAMP* | IRON-SENS | 2 | 0 | 2 | 0.0 |
| *TFR2* | IRON-SENS | 2 | 0 | 2 | 0.0 |
| *TF* | IRON-TRANS | 2 | 0 | 2 | 0.0 |
| *FTL* | IRON-TRANS | 1 | 0 | 1 | 0.0 |
| *CP* | IRON-TRANS | 5 | 0 | 5 | 0.0 |
| *ATP7B* | IRON-TRANS | 6 | 0 | 6 | 0.0 |
| *SERPINA1* | HEPATO-METAB | 15 | 0 | 15 | 0.0 |
| *GBA* | HEPATO-METAB | 3 | 0 | 3 | 0.0 |
| *CFTR* | HEPATO-METAB | 1 | 0 | 1 | 0.0 |
| *HNF1B* | HEPATO-METAB | 1 | 0 | 1 | 0.0 |
| *BCS1L* | HEPATO-METAB | 1 | 0 | 1 | 0.0 |
| *HBB* | ERYTHRO-HEME | 2 | 0 | 2 | 0.0 |
| *ANK1* | ERYTHRO-HEME | 1 | 0 | 1 | 0.0 |
| *SLC4A1* | ERYTHRO-HEME | 1 | 0 | 1 | 0.0 |
| *UROD* | ERYTHRO-HEME | 1 | 0 | 1 | 0.0 |

Gene symbols are presented in bold italics per HUGO Gene Nomenclature Committee guidelines. Genes are ordered by functional pathway: IRON-SENS (Systemic Iron Sensing/Hepcidin Regulation), IRON-TRANS (Iron Transport & Storage), HEPATO-METAB (Hepatic Metabolism & Lysosomal Storage), ERYTHRO-HEME (Erythropoiesis & Heme Biosynthesis). Homozygous indicates patients with biallelic variants in the same gene. Co-occurrence indicates variants in multiple genes, suggesting potential digenic inheritance.

# **Supplementary Table S6. Gene Co-occurrence Patterns Suggestive of Digenic or Oligogenic Inheritance (VUS, Likely Pathogenic, and Pathogenic)**

| **Gene 1** | **Group 1** | **Gene 2** | **Group 2** | **n** |
| --- | --- | --- | --- | --- |
| *HFE* | IRON-SENS | *SERPINA1* | HEPATO-METAB | 9 |
| *HFE* | IRON-SENS | *ATP7B* | IRON-TRANS | 3 |
| *SERPINA1* | HEPATO-METAB | *HBB* | ERYTHRO-HEME | 2 |
| *HFE* | IRON-SENS | *HAMP* | IRON-SENS | 1 |
| *HFE* | IRON-SENS | *FTL* | IRON-TRANS | 1 |
| *HFE* | IRON-SENS | *GBA* | HEPATO-METAB | 1 |
| *HFE* | IRON-SENS | *BCS1L* | HEPATO-METAB | 1 |
| *HFE* | IRON-SENS | *HJV* | IRON-SENS | 1 |
| *HFE* | IRON-SENS | *HBB* | ERYTHRO-HEME | 1 |
| *HAMP* | IRON-SENS | *TFR2* | IRON-SENS | 1 |
| *TF* | IRON-TRANS | *ATP7B* | IRON-TRANS | 1 |
| *TFR2* | IRON-SENS | *ATP7B* | IRON-TRANS | 1 |
| *TF* | IRON-TRANS | *SERPINA1* | HEPATO-METAB | 1 |
| *ATP7B* | IRON-TRANS | *SERPINA1* | HEPATO-METAB | 1 |
| *ATP7B* | IRON-TRANS | *HBB* | ERYTHRO-HEME | 1 |

Gene symbols are presented in bold italics per HUGO Gene Nomenclature Committee guidelines. Genes are ordered by functional pathway: IRON-SENS (Systemic Iron Sensing/Hepcidin Regulation), IRON-TRANS (Iron Transport & Storage), HEPATO-METAB (Hepatic Metabolism & Lysosomal Storage), ERYTHRO-HEME (Erythropoiesis & Heme Biosynthesis). Homozygous indicates patients with biallelic variants in the same gene. Co-occurrence indicates variants in multiple genes, suggesting potential digenic inheritance.

# **Supplementary Table S7. Gene-Level Variant Distribution Restricted to Likely Pathogenic and Pathogenic Variants (N=108)**

| **Gene** | **Functional Group** | **Total Patients** | **Homozygous** | **Heterozygous** | **% Homozygous** |
| --- | --- | --- | --- | --- | --- |
| *HFE* | IRON-SENS | 21 | 3 | 18 | 14.3 |
| *TF* | IRON-TRANS | 1 | 0 | 1 | 0.0 |
| *FTL* | IRON-TRANS | 1 | 0 | 1 | 0.0 |
| *CP* | IRON-TRANS | 2 | 0 | 2 | 0.0 |
| *ATP7B* | IRON-TRANS | 6 | 0 | 6 | 0.0 |
| *SERPINA1* | HEPATO-METAB | 14 | 0 | 14 | 0.0 |
| *GBA* | HEPATO-METAB | 2 | 0 | 2 | 0.0 |
| *HNF1B* | HEPATO-METAB | 1 | 0 | 1 | 0.0 |
| *BCS1L* | HEPATO-METAB | 1 | 0 | 1 | 0.0 |
| *HBB* | ERYTHRO-HEME | 2 | 0 | 2 | 0.0 |
| *ANK1* | ERYTHRO-HEME | 1 | 0 | 1 | 0.0 |
| *SLC4A1* | ERYTHRO-HEME | 1 | 0 | 1 | 0.0 |
| *UROD* | ERYTHRO-HEME | 1 | 0 | 1 | 0.0 |

Gene symbols are presented in bold italics per HUGO Gene Nomenclature Committee guidelines. Genes are ordered by functional pathway: IRON-SENS (Systemic Iron Sensing/Hepcidin Regulation), IRON-TRANS (Iron Transport & Storage), HEPATO-METAB (Hepatic Metabolism & Lysosomal Storage), ERYTHRO-HEME (Erythropoiesis & Heme Biosynthesis). Homozygous indicates patients with biallelic variants in the same gene. Co-occurrence indicates variants in multiple genes, suggesting potential digenic inheritance.

# **Supplementary Table S8. Gene Co-occurrence Patterns Restricted to Likely Pathogenic and Pathogenic Variants**

| **Gene 1** | **Group 1** | **Gene 2** | **Group 2** | **n** |
| --- | --- | --- | --- | --- |
| *HFE* | IRON-SENS | *SERPINA1* | HEPATO-METAB | 4 |
| *SERPINA1* | HEPATO-METAB | *HBB* | ERYTHRO-HEME | 2 |
| *HFE* | IRON-SENS | *ATP7B* | IRON-TRANS | 1 |
| *TF* | IRON-TRANS | *SERPINA1* | HEPATO-METAB | 1 |
| *ATP7B* | IRON-TRANS | *SERPINA1* | HEPATO-METAB | 1 |
| *ATP7B* | IRON-TRANS | *HBB* | ERYTHRO-HEME | 1 |
| *SERPINA1* | HEPATO-METAB | *GBA* | HEPATO-METAB | 1 |

Gene symbols are presented in bold italics per HUGO Gene Nomenclature Committee guidelines. Genes are ordered by functional pathway: IRON-SENS (Systemic Iron Sensing/Hepcidin Regulation), IRON-TRANS (Iron Transport & Storage), HEPATO-METAB (Hepatic Metabolism & Lysosomal Storage), ERYTHRO-HEME (Erythropoiesis & Heme Biosynthesis). Homozygous indicates patients with biallelic variants in the same gene. Co-occurrence indicates variants in multiple genes, suggesting potential digenic inheritance.

# **Supplementary Table S9. Variant-Level Summary Across the Study Population (VUS, Likely Pathogenic, and Pathogenic; N=108)**

| **Variant** | **Gene** | **Total Patients** | **Homozygous** | **Heterozygous** | **% Homozygous** |
| --- | --- | --- | --- | --- | --- |
| *HFE*:c.187C>G | *HFE* | 36 | 4 | 32 | 11.1 |
| *HFE*:c.845G>A | *HFE* | 21 | 3 | 18 | 14.3 |
| *SERPINA1*:c.1096G>A | *SERPINA1* | 7 | 0 | 7 | 0 |
| *SERPINA1*:c.863A>T | *SERPINA1* | 7 | 0 | 7 | 0 |
| *CP*:c.2684G>C | *CP* | 2 | 0 | 2 | 0 |
| *GBA*:c.1448T>C | *GBA* | 2 | 0 | 2 | 0 |
| *HAMP*:c.212G>A | *HAMP* | 2 | 0 | 2 | 0 |
| *ANK1*:c.5504_5507del | *ANK1* | 1 | 0 | 1 | 0 |
| *ATP7B*:c.2605G>A | *ATP7B* | 1 | 0 | 1 | 0 |
| *ATP7B*:c.230dup | *ATP7B* | 1 | 0 | 1 | 0 |
| *ATP7B*:c.1512dup | *ATP7B* | 1 | 0 | 1 | 0 |
| *ATP7B*:c.2978C>T | *ATP7B* | 1 | 0 | 1 | 0 |
| *ATP7B*:c.3275C>T | *ATP7B* | 1 | 0 | 1 | 0 |
| *ATP7B*:c.3053C>T | *ATP7B* | 1 | 0 | 1 | 0 |
| *BCS1L*:c.460+2T>C | *BCS1L* | 1 | 0 | 1 | 0 |
| *CFTR*:c.2991G>C | *CFTR* | 1 | 0 | 1 | 0 |
| *CP*:c.2520_2523del | *CP* | 1 | 0 | 1 | 0 |
| *CP*:c.2476_2495del | *CP* | 1 | 0 | 1 | 0 |
| *CP*:c.2799C>A | *CP* | 1 | 0 | 1 | 0 |
| *FTL*:c.89C>T | *FTL* | 1 | 0 | 1 | 0 |
| *GBA*:c.247C>T | *GBA* | 1 | 0 | 1 | 0 |
| *GBA*:c.115+3G>A | *GBA* | 1 | 0 | 1 | 0 |
| *GBA*:c.115+4A>C | *GBA* | 1 | 0 | 1 | 0 |
| *HBB*:c.118C>T | *HBB* | 1 | 0 | 1 | 0 |
| *HBB*:c.126_129del | *HBB* | 1 | 0 | 1 | 0 |
| *HFE*:c.710T>G | *HFE* | 1 | 0 | 1 | 0 |
| *HFE*:c.854A>C | *HFE* | 1 | 0 | 1 | 0 |
| *HJV*:c.904G>A | *HJV* | 1 | 0 | 1 | 0 |
| NC_000017.10:g.(34892941_36104886)del† | *HNF1B* | 1 | 0 | 1 | 0 |
| *SERPINA1*:c.922G>T | *SERPINA1* | 1 | 0 | 1 | 0 |
| *SLC4A1*:c.448C>T | *SLC4A1* | 1 | 0 | 1 | 0 |
| *TF*:c.1203+1G>T | *TF* | 1 | 0 | 1 | 0 |
| *TF*:c.1925G>A | *TF* | 1 | 0 | 1 | 0 |
| *TFR2*:c.1118G>A | *TFR2* | 1 | 0 | 1 | 0 |
| *TFR2*:c.1513G>A | *TFR2* | 1 | 0 | 1 | 0 |
| *UROD*:c.390G>T | *UROD* | 1 | 0 | 1 | 0 |

Variants are presented as “GENE:HGVS nomenclature”. HMZ = Homozygous; HTZ = Heterozygous. Digenic indicates variants in different genes occurring in the same patient, suggesting potential digenic inheritance patterns.

†The *HNF1B* variant is a 1.21 Mb deletion at 17q12 (seq[GRCh37] del(17)(q12); chr17:34892941-36104886; NC_000017.10:g.(34892941_36104886)del) encompassing 17 loci: *HNF1B*, *PIGW*, *MRM1*, *DHRS11*, *LHX1*, *C17orf78*, *DDX52*, *ACACA*, *SNORA90*, *GGNBP2*, *TADA2A*, *SYNRG*, *AATF*, *MIR378J*, *DUSP14*, *LHX1-DT*, and *MIR2909*. The deletion was confirmed using chromosomal microarray analysis in an independent ISO 15189-accredited laboratory. The phenotypic effect was attributed to *HNF1B* haploinsufficiency based on ClinGen Haploinsufficiency score of 3, dominant inheritance pattern associated with hepatic manifestations (OMIM #614527, GeneReviews NBK401562, and reference ^18^), and high probability of loss-of-function intolerance (pLI = 1; o/e LOF upper = 0.17; DECIPHER HI score = 0.76). Other genes in the region have no established disease association, follow autosomal recessive inheritance (*PIGW*, *ACACA*), or lack evidence of haploinsufficiency.

# **Supplementary Table S10. Variant Co-occurrence Patterns (VUS, Likely Pathogenic, and Pathogenic)**

| **Variant 1** | **Gene 1** | **Variant 2** | **Gene 2** | **Co-occurrence (n)** | **Relationship** |
| --- | --- | --- | --- | --- | --- |
| *HFE*:c.187C>G | *HFE* | *HFE*:c.845G>A | *HFE* | 7 | Same gene |
| *HFE*:c.187C>G | *HFE* | *SERPINA1*:c.863A>T | *SERPINA1* | 5 | Digenic† |
| *HFE*:c.845G>A | *HFE* | *SERPINA1*:c.863A>T | *SERPINA1* | 2 | Digenic† |
| *HFE*:c.187C>G | *HFE* | *SERPINA1*:c.1096G>A | *SERPINA1* | 2 | Digenic† |
| *HFE*:c.845G>A | *HFE* | *SERPINA1*:c.1096G>A | *SERPINA1* | 2 | Digenic† |
| *ATP7B*:c.1512dup | *ATP7B* | *HBB*:c.126_129del | *HBB* | 1 | Digenic |
| *ATP7B*:c.3053C>T | *ATP7B* | *HFE*:c.845G>A | *HFE* | 1 | Digenic |
| *ATP7B*:c.1512dup | *ATP7B* | *HFE*:c.187C>G | *HFE* | 1 | Digenic |
| *ATP7B*:c.2605G>A | *ATP7B* | *HFE*:c.187C>G | *HFE* | 1 | Digenic |
| *ATP7B*:c.1512dup | *ATP7B* | *SERPINA1*:c.1096G>A | *SERPINA1* | 1 | Digenic |
| *ATP7B*:c.2978C>T | *ATP7B* | *TF*:c.1925G>A | *TF* | 1 | Digenic |
| *ATP7B*:c.3275C>T | *ATP7B* | *TFR2*:c.1513G>A | *TFR2* | 1 | Digenic |
| *BCS1L*:c.460+2T>C | *BCS1L* | *HFE*:c.187C>G | *HFE* | 1 | Digenic |
| *FTL*:c.89C>T | *FTL* | *HFE*:c.187C>G | *HFE* | 1 | Digenic |
| *GBA*:c.1448T>C | *GBA* | *GBA*:c.247C>T | *GBA* | 1 | Same gene |
| *GBA*:c.115+3G>A | *GBA* | *GBA*:c.115+4A>C | *GBA* | 1 | Same gene |
| *GBA*:c.1448T>C | *GBA* | *HFE*:c.187C>G | *HFE* | 1 | Digenic |
| *GBA*:c.247C>T | *GBA* | *HFE*:c.187C>G | *HFE* | 1 | Digenic |
| *GBA*:c.1448T>C | *GBA* | *SERPINA1*:c.863A>T | *SERPINA1* | 1 | Digenic |
| *HAMP*:c.212G>A | *HAMP* | *HFE*:c.187C>G | *HFE* | 1 | Digenic |
| *HAMP*:c.212G>A | *HAMP* | *TFR2*:c.1118G>A | *TFR2* | 1 | Digenic |
| *HBB*:c.126_129del | *HBB* | *HFE*:c.187C>G | *HFE* | 1 | Digenic |
| *HBB*:c.126_129del | *HBB* | *SERPINA1*:c.1096G>A | *SERPINA1* | 1 | Digenic |
| *HBB*:c.118C>T | *HBB* | *SERPINA1*:c.1096G>A | *SERPINA1* | 1 | Digenic |
| *HFE*:c.710T>G | *HFE* | *HFE*:c.845G>A | *HFE* | 1 | Same gene |
| *HFE*:c.187C>G | *HFE* | *HJV*:c.904G>A | *HJV* | 1 | Digenic |
| *HFE*:c.187C>G | *HFE* | *SERPINA1*:c.922G>T | *SERPINA1* | 1 | Digenic |
| *SERPINA1*:c.863A>T | *SERPINA1* | *TF*:c.1203+1G>T | *TF* | 1 | Digenic |

Variants are presented as "GENE:HGVS nomenclature"; Digenic indicates variants in different genes occurring in the same patient; A single patient may exhibit more than one digenic combination.

A total of 15 patients carried heterozygous *SERPINA1* variants: 7 carried PI*Z (p.Glu366Lys), 7 carried PI*S (p.Glu288Val), and 1 carried PI*M1Lyon (p.Ala308Ser). Among PI*Z carriers, four exhibited digenic combinations with *HFE*, *HBB*, or *ATP7B*, whereas three carried the PI*Z variant alone. All PI*S carriers exhibited digenic combinations, most frequently with *HFE*, but also with *GBA* and *TF*.

†Based on Hardy-Weinberg equilibrium and observed allele frequencies in the gnomAD exome dataset (European Non-Finnish population), the expected frequencies for an individual to carry heterozygous variants in both *HFE* and *SERPINA1* are as follows: *HFE* p.His63Asp with *SERPINA1* PI*S, approximately 1.76% (1 in 57); *HFE* p.His63Asp with *SERPINA1* PI*Z, approximately 0.87% (1 in 115); *HFE* p.Cys282Tyr with *SERPINA1* PI*S, approximately 0.77% (1 in 130); and *HFE* p.Cys282Tyr with *SERPINA1* PI*Z, approximately 0.38% (1 in 263).

# **Supplementary Table S11. Variant-Level Summary Restricted to Likely Pathogenic and Pathogenic Variants (N=108)**

| **Variant** | **Gene** | **Total** | **HMZ** | **HTZ** | **% HMZ** |
| --- | --- | --- | --- | --- | --- |
| *HFE*:c.845G>A | *HFE* | 21 | 3 | 18 | 14.3 |
| *SERPINA1*:c.1096G>A | *SERPINA1* | 7 | 0 | 7 | 0.0 |
| *SERPINA1*:c.863A>T | *SERPINA1* | 7 | 0 | 7 | 0.0 |
| *GBA*:c.1448T>C | *GBA* | 2 | 0 | 2 | 0.0 |
| *ANK1*:c.5504_5507del | *ANK1* | 1 | 0 | 1 | 0.0 |
| *ATP7B*:c.1512dup | *ATP7B* | 1 | 0 | 1 | 0.0 |
| *ATP7B*:c.3053C>T | *ATP7B* | 1 | 0 | 1 | 0.0 |
| *ATP7B*:c.2978C>T | *ATP7B* | 1 | 0 | 1 | 0.0 |
| *ATP7B*:c.2605G>A | *ATP7B* | 1 | 0 | 1 | 0.0 |
| *ATP7B*:c.230dup | *ATP7B* | 1 | 0 | 1 | 0.0 |
| *ATP7B*:c.3275C>T | *ATP7B* | 1 | 0 | 1 | 0.0 |
| *BCS1L*:c.460+2T>C | *BCS1L* | 1 | 0 | 1 | 0.0 |
| *CP*:c.2520_2523del | *CP* | 1 | 0 | 1 | 0.0 |
| *CP*:c.2476_2495del | *CP* | 1 | 0 | 1 | 0.0 |
| *FTL*:c.89C>T | *FTL* | 1 | 0 | 1 | 0.0 |
| *GBA*:c.247C>T | *GBA* | 1 | 0 | 1 | 0.0 |
| *HBB*:c.118C>T | *HBB* | 1 | 0 | 1 | 0.0 |
| *HBB*:c.126_129del | *HBB* | 1 | 0 | 1 | 0.0 |
| NC_000017.10:g.(34892941_36104886)del† | *HNF1B* | 1 | 0 | 1 | 0.0 |
| *SLC4A1*:c.448C>T | *SLC4A1* | 1 | 0 | 1 | 0.0 |
| *TF*:c.1203+1G>T | *TF* | 1 | 0 | 1 | 0.0 |
| *UROD*:c.390G>T | *UROD* | 1 | 0 | 1 | 0.0 |

Variants are presented as “GENE:HGVS nomenclature”. HMZ = Homozygous; HTZ = Heterozygous. Digenic indicates variants in different genes occurring in the same patient, suggesting potential digenic inheritance patterns.

†The *HNF1B* variant is a 1.21 Mb deletion at 17q12 (seq[GRCh37] del(17)(q12); chr17:34892941-36104886; NC_000017.10:g.(34892941_36104886)del) encompassing 17 loci: *HNF1B*, *PIGW*, *MRM1*, *DHRS11*, *LHX1*, *C17orf78*, *DDX52*, *ACACA*, *SNORA90*, *GGNBP2*, *TADA2A*, *SYNRG*, *AATF*, *MIR378J*, *DUSP14*, *LHX1-DT*, and *MIR2909*. The deletion was confirmed using chromosomal microarray analysis in an independent ISO 15189-accredited laboratory. The phenotypic effect was attributed to *HNF1B* haploinsufficiency based on ClinGen Haploinsufficiency score of 3, dominant inheritance pattern associated with hepatic manifestations (OMIM #614527, GeneReviews NBK401562, and reference ^18^), and high probability of loss-of-function intolerance (pLI = 1; o/e LOF upper = 0.17; DECIPHER HI score = 0.76). Other genes in the region have no established disease association, follow autosomal recessive inheritance (*PIGW*, *ACACA*), or lack evidence of haploinsufficiency.

# **Supplementary Table S12. Variant Co-occurrence Patterns Restricted to Likely Pathogenic and Pathogenic Variants**

| **Variant 1** | **Variant 2** | **n** | **Genes** | **Type** |
| --- | --- | --- | --- | --- |
| *HFE*:c.845G>A | *SERPINA1*:c.1096G>A | 2 | *HFE/SERPINA1* | Digenic |
| *HFE*:c.845G>A | *SERPINA1*:c.863A>T | 2 | *HFE/SERPINA1* | Digenic |
| *ATP7B*:c.1512dup | *SERPINA1*:c.1096G>A | 1 | *ATP7B/SERPINA1* | Digenic |
| *ATP7B*:c.1512dup | *HBB*:c.126_129del | 1 | *ATP7B/HBB* | Digenic |
| *ATP7B*:c.3053C>T | *HFE*:c.845G>A | 1 | *ATP7B/HFE* | Digenic |
| *GBA*:c.1448T>C | *GBA*:c.247C>T | 1 | *GBA/GBA* | Same gene |
| *HBB*:c.118C>T | *SERPINA1*:c.1096G>A | 1 | *HBB/SERPINA1* | Digenic |
| *GBA*:c.1448T>C | *SERPINA1*:c.863A>T | 1 | *GBA/SERPINA1* | Digenic |
| *HBB*:c.126_129del | *SERPINA1*:c.1096G>A | 1 | *HBB/SERPINA1* | Digenic |
| *SERPINA1*:c.863A>T | *TF*:c.1203+1G>T | 1 | *SERPINA1/TF* | Digenic |

Variants are presented as “GENE:HGVS nomenclature”. HMZ = Homozygous; HTZ = Heterozygous. Digenic indicates variants in different genes occurring in the same patient, suggesting potential digenic inheritance patterns.

# **Supplementary Table S13. Patient Classification by Functional Gene Pathway**

| **Variable** | **Category** | **No. (%)** |
| --- | --- | --- |
| **Variants classified as VUS/LP/P (n=72)** |  |  |
| Single functional group (mutually exclusive) |  |  |
|  | IRON-SENS | 38 (52.8) |
|  | IRON-TRANS | 7 (9.7) |
|  | HEPATO-METAB | 7 (9.7) |
|  | ERYTHRO-HEME | 3 (4.2) |
| Multi-group combinations (mutually exclusive) |  |  |
|  | IRON-SENS + HEPATO-METAB | 10 (13.9) |
|  | IRON-SENS + IRON-TRANS | 4 (5.6) |
|  | HEPATO-METAB + ERYTHRO-HEME | 1 (1.4) |
|  | IRON-TRANS + HEPATO-METAB | 1 (1.4) |
|  | IRON-SENS + IRON-TRANS + HEPATO-METAB + ERYTHRO-HEME | 1 (1.4) |
| **Variants classified as LP/P only (n=44)** |  |  |
| Single functional group (mutually exclusive) |  |  |
|  | IRON-SENS | 16 (36.4) |
|  | IRON-TRANS | 7 (15.9) |
|  | HEPATO-METAB | 10 (22.7) |
|  | ERYTHRO-HEME | 3 (6.8) |
| Multi-group combinations (mutually exclusive) |  |  |
|  | IRON-SENS + HEPATO-METAB | 4 (9.1) |
|  | HEPATO-METAB + ERYTHRO-HEME | 1 (2.3) |
|  | IRON-SENS + IRON-TRANS | 1 (2.3) |
|  | IRON-TRANS + HEPATO-METAB | 1 (2.3) |
|  | IRON-TRANS + HEPATO-METAB + ERYTHRO-HEME | 1 (2.3) |

For functional gene group classification, patients are counted mutually exclusively: if variants map to ≥2 functional groups, patients are reported under the corresponding multi-group combination instead of being counted in each group separately. Functional gene groups (genes reported once): IRON-SENS (*HFE*, *HJV*, *HAMP*, *TFR2*); IRON-TRANS (*TF*, *CP*, *FTL*, *ATP7B*); HEPATO-METAB (*SERPINA1*, *HNF1B*, *GBA*, *CFTR*, *BCS1L*); ERYTHRO-HEME (*HBB*, *ANK1*, *UROD*, *SLC4A1*).

Abbreviations: LP, likely pathogenic; P, pathogenic; VUS, variant of uncertain significance

# **SUPPLEMENTARY FIGURES**

# **Supplementary Figure S1. Gene Co-occurrence Heatmap Restricted to Likely Pathogenic and Pathogenic Variants**


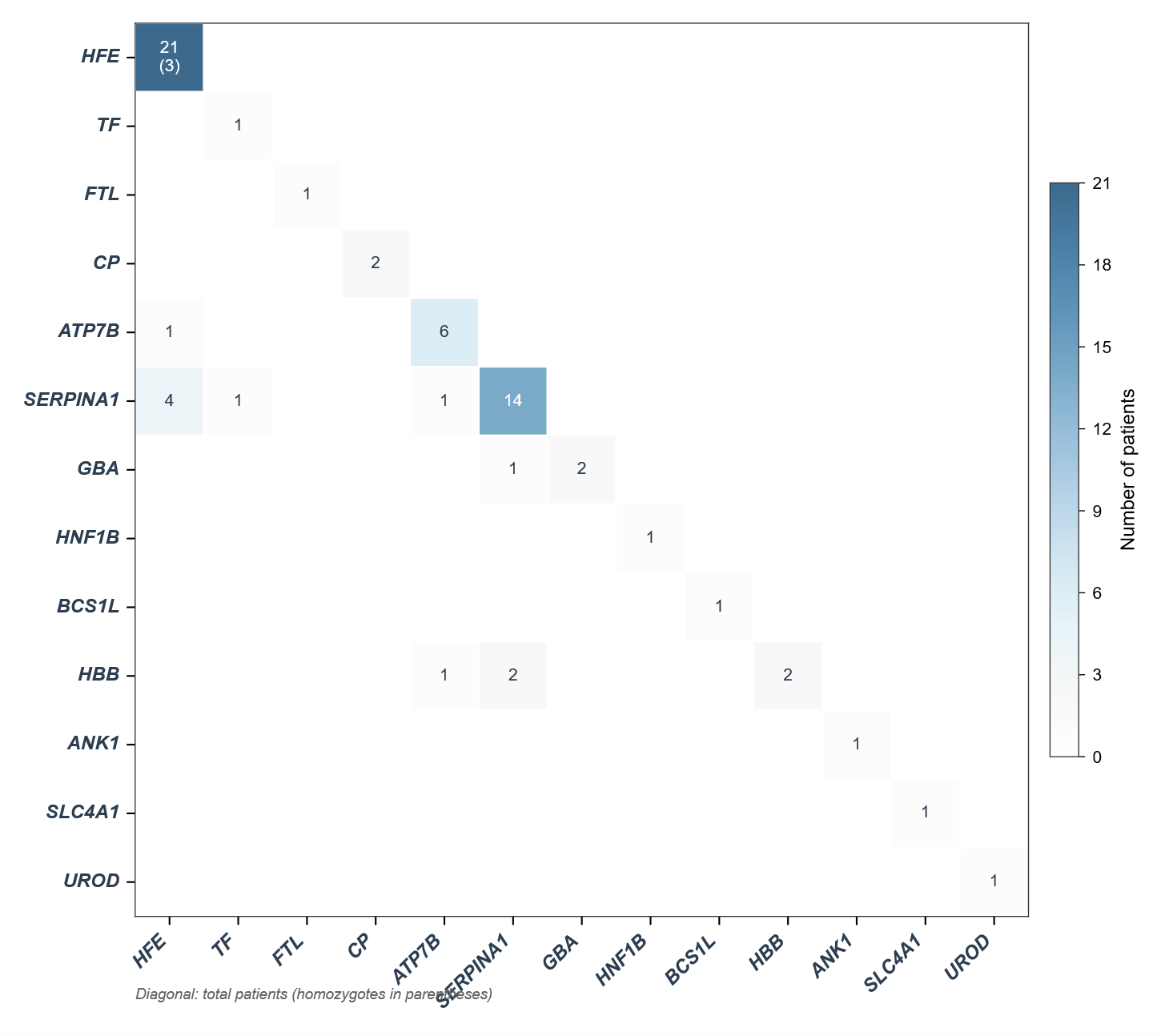
Heatmap displaying gene co-occurrence patterns among patients carrying LP or P variants only (LP, likely pathogenic; P, pathogenic). Diagonal values indicate the total number of patients with variants in each gene (patients with two variants in the same gene are counted once), with homozygous patients shown in parentheses. Off-diagonal values represent the number of patients harboring variants in both genes. *HFE* was involved in 21 patients (3 homozygous), followed by *SERPINA1* (n=14), *ATP7B* (n=6), *CP* (n=2), *GBA* (n=2), and *HBB* (n=2). The most frequent digenic combinations involved *HFE* and *SERPINA1* (n=4), *SERPINA1* and *HBB* (n=2), and *HFE* and *ATP7B* (n=1). Gene symbols are presented according to HUGO Gene Nomenclature Committee guidelines.

# **Supplementary Figure S2. Functional Pathway Distribution Including All Retained Variants**


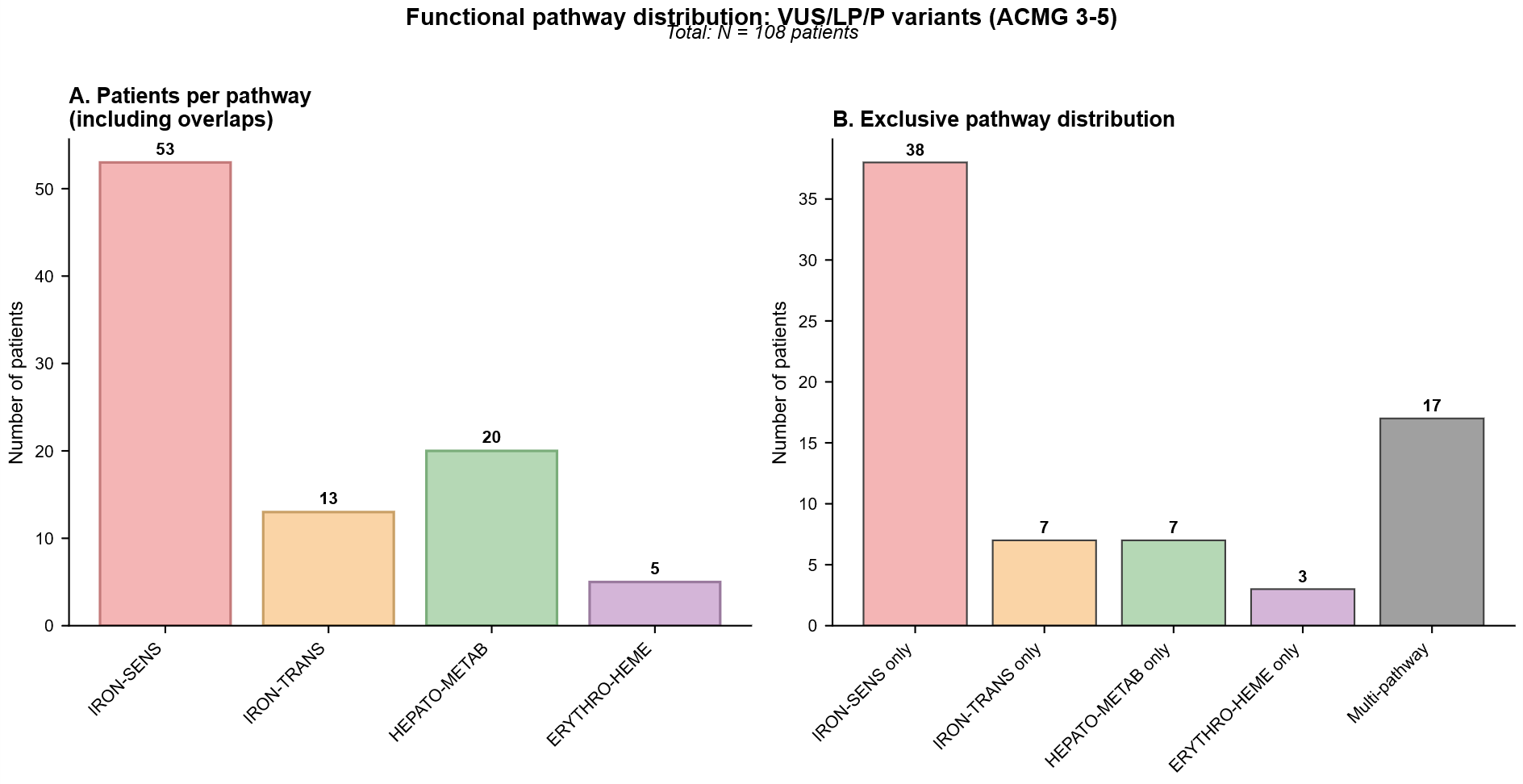


Bar charts displaying patient distribution across functional pathway groups among patients carrying VUS, LP, or P variants (VUS, variant of uncertain significance; LP, likely pathogenic; P, pathogenic). **(A)** Total number of patients per pathway, including those with variants in multiple pathways. **(B)** Exclusive pathway distribution showing patients belonging to a single pathway only, patients with multi-pathway involvement, and patients without retained variants (negative). IRON-SENS, systemic iron sensing and hepcidin regulation; IRON-TRANS, iron transport and storage; HEPATO-METAB, hepatic metabolism and lysosomal storage; ERYTHRO-HEME, erythropoiesis and heme biosynthesis.

# **Supplementary Figure S3. Pairwise Pathway Overlaps Including All Retained Variants**


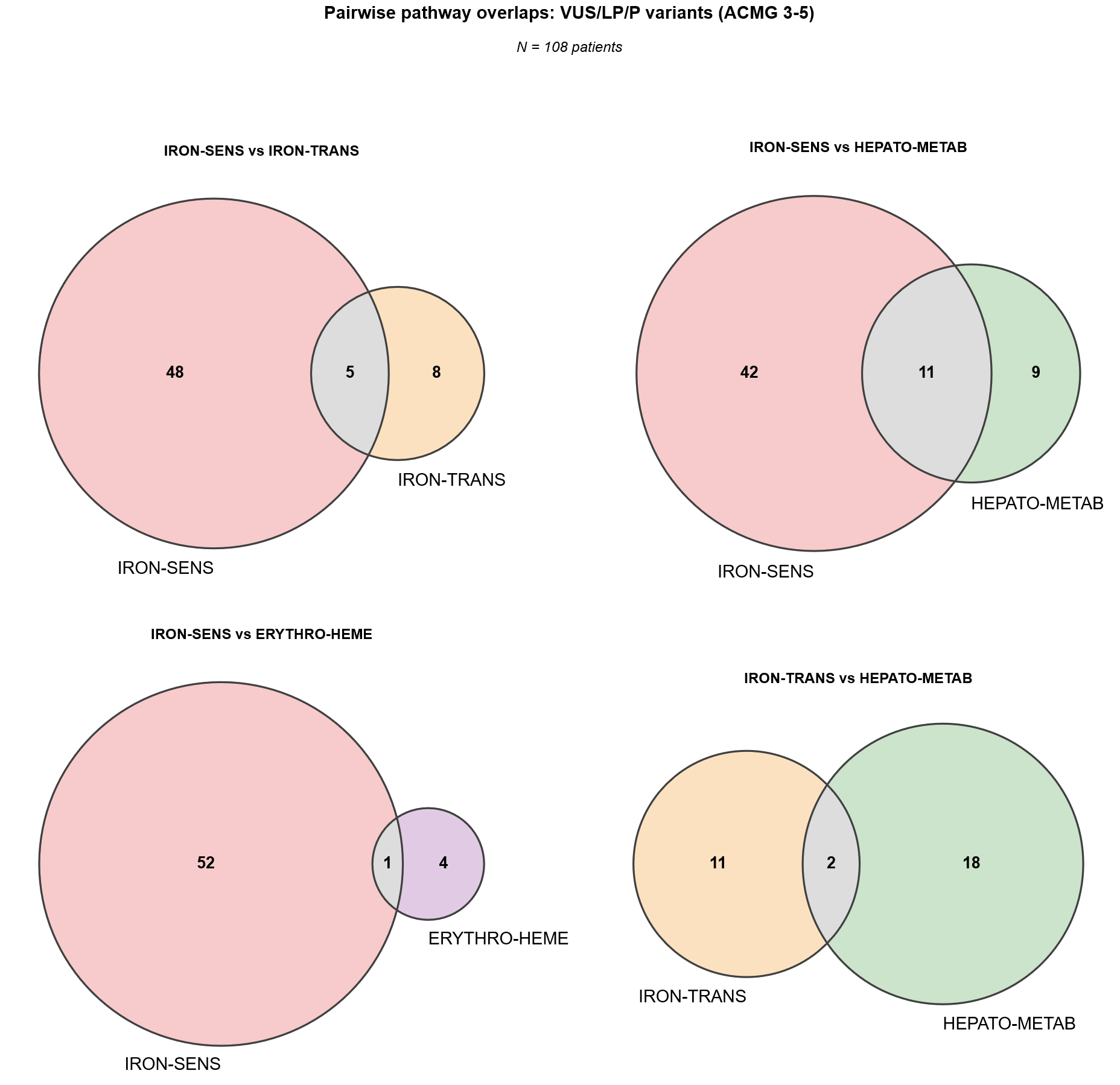


Pairwise Venn diagrams illustrating patient overlap between functional pathway groups among patients carrying VUS, LP, or P variants (VUS, variant of uncertain significance; LP, likely pathogenic; P, pathogenic). Each diagram displays the number of patients exclusive to each pathway and the number shared between both pathways. Four key pairwise comparisons are shown: IRON-SENS versus IRON-TRANS, IRON-SENS versus HEPATO-METAB, IRON-SENS versus ERYTHRO-HEME, and IRON-TRANS versus HEPATO-METAB. IRON-SENS, systemic iron sensing and hepcidin regulation; IRON-TRANS, iron transport and storage; HEPATO-METAB, hepatic metabolism and lysosomal storage; ERYTHRO-HEME, erythropoiesis and heme biosynthesis.

# **Supplementary Figure S4. Functional Pathway Distribution Restricted to Likely Pathogenic and Pathogenic Variants**


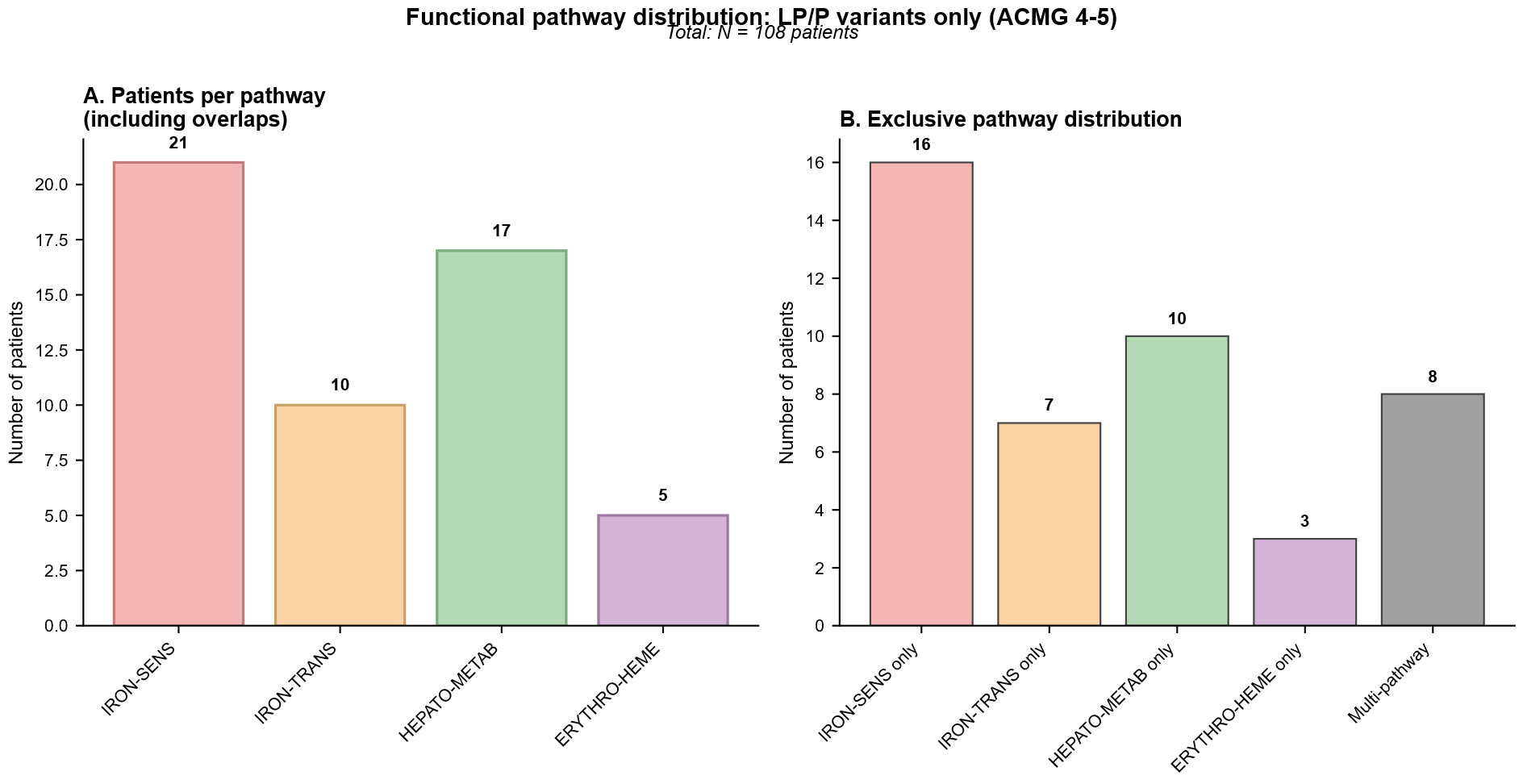


Bar charts displaying patient distribution across functional pathway groups among patients carrying LP or P variants only (LP, likely pathogenic; P, pathogenic). **(A)** Total number of patients per pathway, including those with variants in multiple pathways. **(B)** Exclusive pathway distribution showing patients belonging to a single pathway only, patients with multi-pathway involvement, and patients without LP or P variants (negative). IRON-SENS, systemic iron sensing and hepcidin regulation; IRON-TRANS, iron transport and storage; HEPATO-METAB, hepatic metabolism and lysosomal storage; ERYTHRO-HEME, erythropoiesis and heme biosynthesis.

# **Supplementary Figure S5. Pairwise Pathway Overlaps Restricted to Likely Pathogenic and Pathogenic Variants**


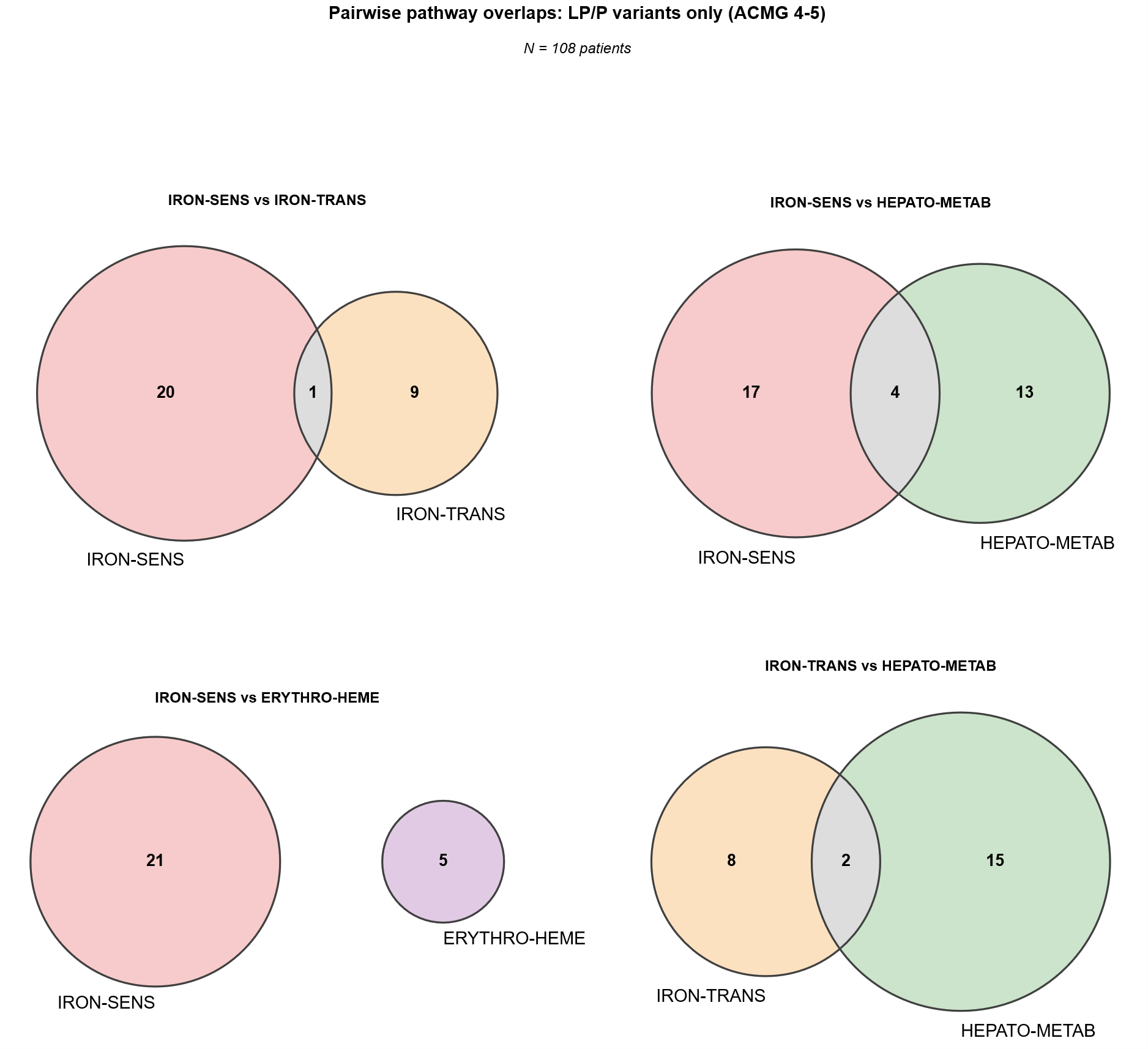


Pairwise Venn diagrams illustrating patient overlap between functional pathway groups among patients carrying LP or P variants only (LP, likely pathogenic; P, pathogenic). Each diagram displays the number of patients exclusive to each pathway and the number shared between both pathways. Four key pairwise comparisons are shown: IRON-SENS versus IRON-TRANS, IRON-SENS versus HEPATO-METAB, IRON-SENS versus ERYTHRO-HEME, and IRON-TRANS versus HEPATO-METAB. IRON-SENS, systemic iron sensing and hepcidin regulation; IRON-TRANS, iron transport and storage; HEPATO-METAB, hepatic metabolism and lysosomal storage; ERYTHRO-HEME, erythropoiesis and heme biosynthesis.

# **Supplementary Figure S6. Iron Metabolism Parameters by Functional Pathway Including All Retained Variants**


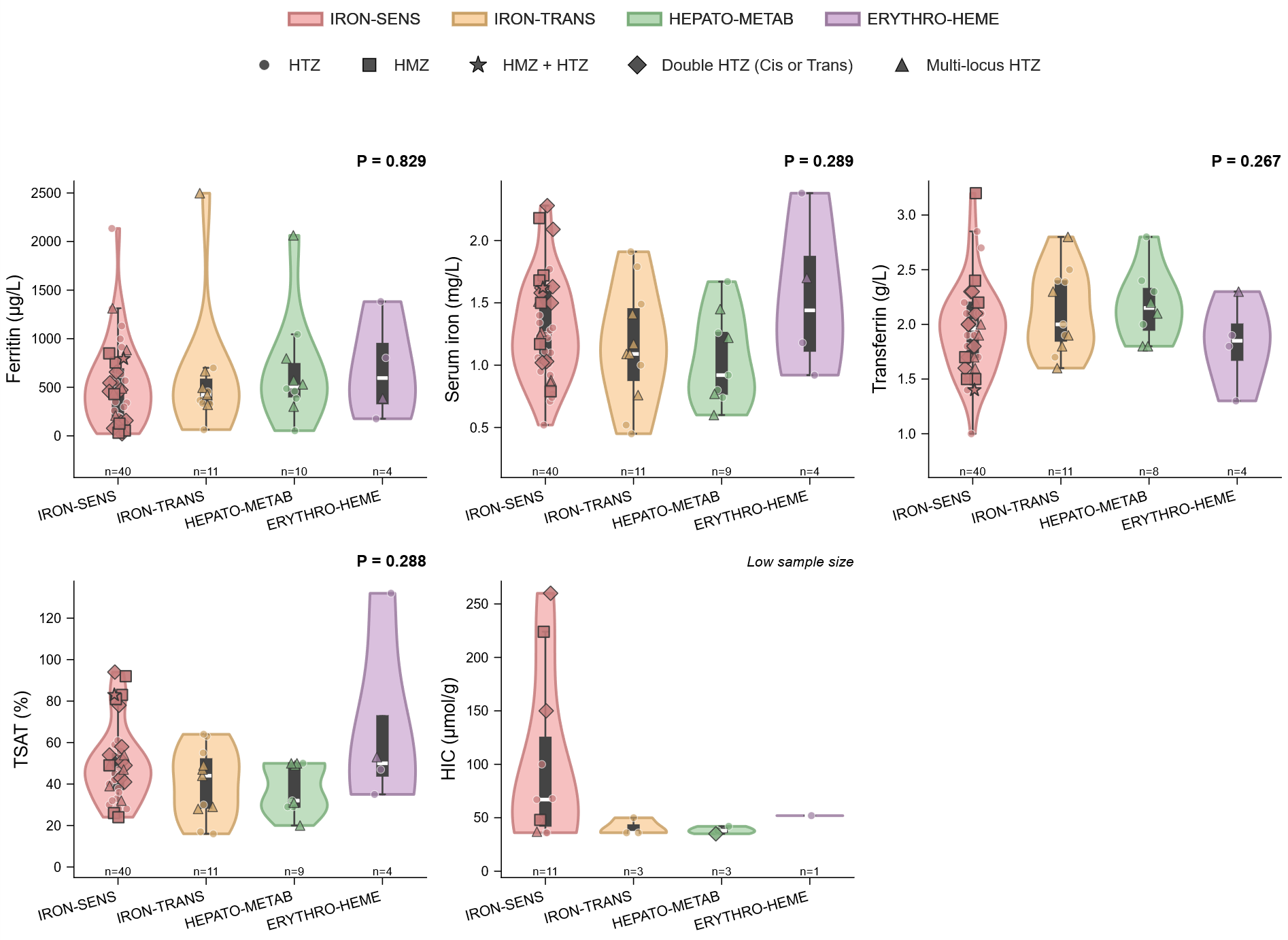


Violin plots showing the distribution of iron metabolism parameters across four functional pathway groups in patients with any retained variant (VUS, LP, or P; VUS, variant of uncertain significance; LP, likely pathogenic; P, pathogenic). Each plot displays the median (white line), interquartile range (black box), and individual data points stratified by zygosity: heterozygous (HTZ, circles), homozygous (HMZ, squares), homozygous with additional heterozygous variant (HMZ + HTZ, stars), double heterozygous in the same gene in cis or trans configuration (diamonds), and multi-locus heterozygous (triangles). Sample sizes are indicated below each group. P values are from Kruskal-Wallis tests. When including all retained variants, serum ferritin concentrations (P = 0.829), serum iron (P = 0.289), transferrin (P = 0.267), and transferrin saturation (P = 0.288) did not differ significantly across pathway groups. Statistical comparison of hepatic iron concentration was not performed owing to sparse data precluding valid inference (IRON-SENS, n=11; IRON-TRANS, n=3; HEPATO-METAB, n=3; ERYTHRO-HEME, n=1). IRON-SENS, systemic iron sensing and hepcidin regulation (*HFE*, *HJV*, *HAMP*, *TFR2*); IRON-TRANS, iron transport and storage (*TF*, *CP*, *FTL*, *ATP7B*); HEPATO-METAB, hepatic metabolism and lysosomal storage (*SERPINA1*, *HNF1B*, *GBA*, *CFTR*, *BCS1L*); ERYTHRO-HEME, erythropoiesis and heme biosynthesis (*HBB*, *ANK1*, *UROD*, *SLC4A1*)

# **Supplementary Figure S7. Iron Metabolism Parameters in Patients With Digenic *HFE*/*SERPINA1*, *HFE*/*ATP7B*, and *SERPINA1*/*HBB* Combinations Compared With Monogenic *HFE* Genotypes**


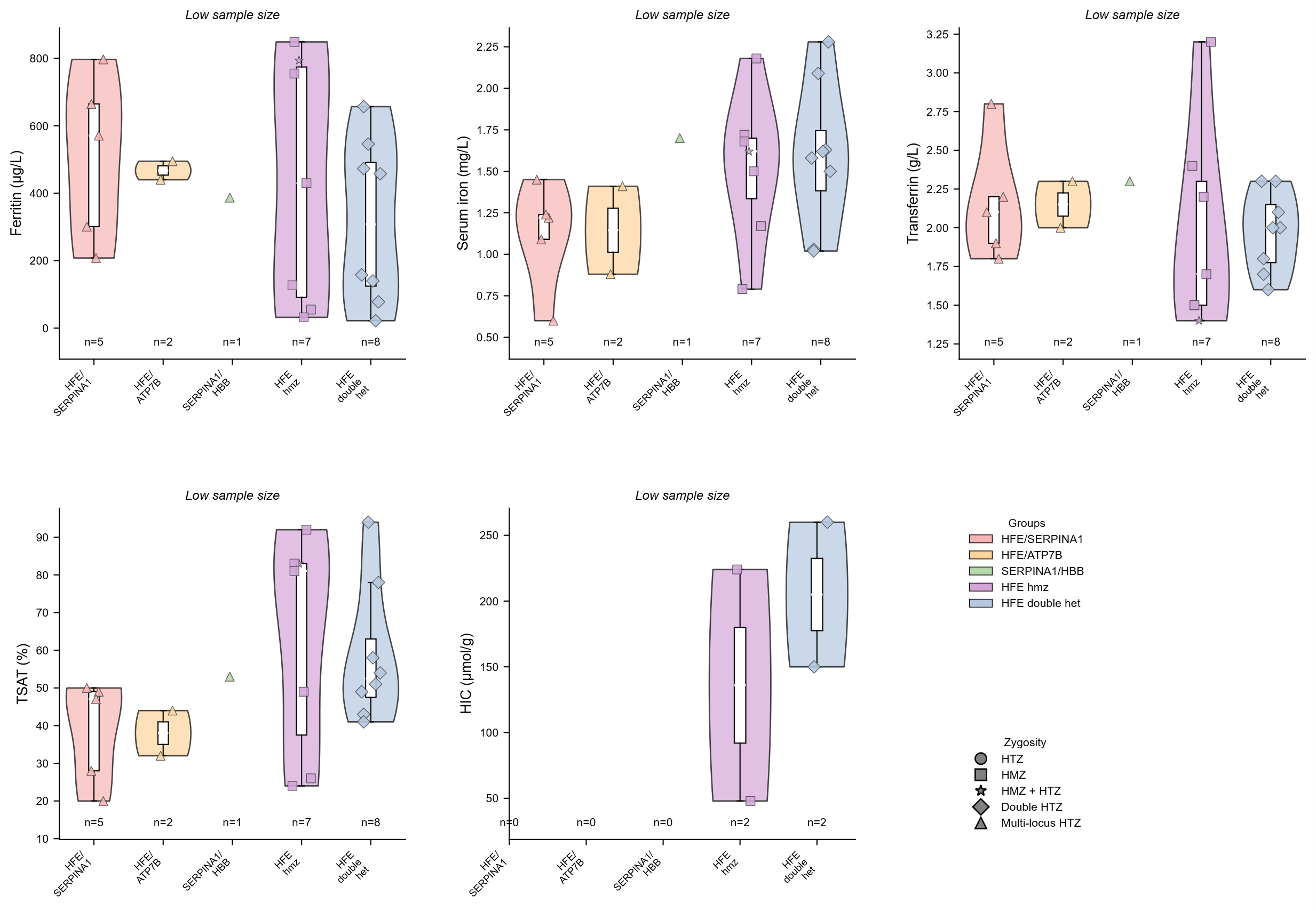


Violin plots displaying the distribution of iron metabolism parameters across selected genotypic combinations. Digenic groups include patients with co-occurring variants in *HFE* and *SERPINA1* (n=5; *HFE* p.Cys282Tyr or p.His63Asp with *SERPINA1* p.Glu366Lys [PI*Z] or p.Glu288Val [PI*S]), *HFE* and *ATP7B* (n=2; *HFE* p.Cys282Tyr or p.His63Asp with *ATP7B* p.Ala1018Val or p.Pro505Leufs*6), and *SERPINA1* and *HBB* (n=1; *SERPINA1* p.Glu366Lys with *HBB* p.Gln40Ter). Monogenic *HFE* groups comprise patients homozygous for *HFE* variants (HFE HMZ, n=7; including p.Cys282Tyr homozygotes and p.His63Asp homozygotes) and patients with double heterozygosity for *HFE* p.Cys282Tyr and p.His63Asp (HFE double HTZ, n=8). Each plot displays individual data points stratified by zygosity: heterozygous (HTZ, circles), homozygous (HMZ, squares), combined homozygous and heterozygous (HMZ + HTZ, stars), double heterozygous in the same gene (diamonds), and multi-locus heterozygous (triangles). Sample sizes are indicated below each group and may vary across parameters owing to missing data. Hepatic iron concentration data were available for only a subset of patients in the HFE HMZ (n=2) and HFE double HTZ (n=2) groups, with no data available for the digenic groups. Statistical comparisons were not performed owing to the small sample sizes, which preclude valid inference. Abbreviations: HIC, hepatic iron concentration; HMZ, homozygous; HTZ, heterozygous; TSAT, transferrin saturation.

**REFERENCES**

1. Alix T, Chery C, Josse T, et al. Predictors of the utility of clinical exome sequencing as a first-tier genetic test in patients with Mendelian phenotypes: results from a referral center study on 603 consecutive cases. *Hum Genomics.* 2023;17(1):5.

2. Mergnac JP, Wiedemann A, Chery C, et al. Diagnostic yield of clinical exome sequencing as a first-tier genetic test for the diagnosis of genetic disorders in pediatric patients: results from a referral center study. *Hum Genet.* 2022;141(7):1269-1278.

3. Silva Rodriguez M, Mulot M, Chery C, et al. Exome-based genotype-first reverse phenotyping using structured electronic health record data identifies novel SERPINA1 variants associated with liver markers and demonstrates a dominant effect for specific variants on liver phenotype. *Hepatol Res.* 2025;55(7):1075-1092.

4. Gueant JL, Chery C, Oussalah A, et al. APRDX1 mutant allele causes a MMACHC secondary epimutation in cblC patients. *Nat Commun.* 2018;9(1):67.

5. Renard E, Chéry C, Oussalah A, et al. Exome sequencing of cases with neural tube defects identifies candidate genes involved in one-carbon/vitamin B12 metabolisms and Sonic Hedgehog pathway. *Hum Genet.* 2019;138(7):703-713.

6. Wiedemann A, Chery C, Coelho D, et al. Mutations in MTHFR and POLG impaired activity of the mitochondrial respiratory chain in 46-year-old twins with spastic paraparesis. *Journal of human genetics.* 2020;65(2):91-98.

7. Mergnac JP, Wiedemann A, Chery C, et al. Diagnostic yield of clinical exome sequencing as a first-tier genetic test for the diagnosis of genetic disorders in pediatric patients: results from a referral center study. *Hum Genet 2021 DOI: 101007/s00439-021-02358-0.* 2021.

8. Van der Auwera GA, Carneiro MO, Hartl C, et al. From FastQ data to high confidence variant calls: the Genome Analysis Toolkit best practices pipeline. *Curr Protoc Bioinformatics.* 2013;43:11 10 11-11 10 33.

9. Singleton MV, Guthery SL, Voelkerding KV, et al. Phevor combines multiple biomedical ontologies for accurate identification of disease-causing alleles in single individuals and small nuclear families. *Am J Hum Genet.* 2014;94(4):599-610.

10. Schwarz JM, Cooper DN, Schuelke M, Seelow D. MutationTaster2: mutation prediction for the deep-sequencing age. *Nat Methods.* 2014;11(4):361-362.

11. Shihab HA, Gough J, Cooper DN, et al. Predicting the functional, molecular, and phenotypic consequences of amino acid substitutions using hidden Markov models. *Hum Mutat.* 2013;34(1):57-65.

12. Dong C, Wei P, Jian X, et al. Comparison and integration of deleteriousness prediction methods for nonsynonymous SNVs in whole exome sequencing studies. *Hum Mol Genet.* 2015;24(8):2125-2137.

13. Kumar P, Henikoff S, Ng PC. Predicting the effects of coding non-synonymous variants on protein function using the SIFT algorithm. *Nat Protoc.* 2009;4(7):1073-1081.

14. Choi Y, Chan AP. PROVEAN web server: a tool to predict the functional effect of amino acid substitutions and indels. *Bioinformatics.* 2015;31(16):2745-2747.

15. Quang D, Chen Y, Xie X. DANN: a deep learning approach for annotating the pathogenicity of genetic variants. *Bioinformatics.* 2015;31(5):761-763.

16. Richards S, Aziz N, Bale S, et al. Standards and guidelines for the interpretation of sequence variants: a joint consensus recommendation of the American College of Medical Genetics and Genomics and the Association for Molecular Pathology. *Genet Med.* 2015;17(5):405-424.

17. Landrum MJ, Lee JM, Benson M, et al. ClinVar: improving access to variant interpretations and supporting evidence. *Nucleic Acids Res.* 2018;46(D1):D1062-D1067.

18. Dubois-Laforgue D, Cornu E, Saint-Martin C, et al. Diabetes, Associated Clinical Spectrum, Long-term Prognosis, and Genotype/Phenotype Correlations in 201 Adult Patients With Hepatocyte Nuclear Factor 1B (HNF1B) Molecular Defects. *Diabetes Care.* 2017;40(11):1436-1443.
